# Gut microbial metabolite indole-3-propionic acid inhibits inflammation and restores blood-milk barrier in *S. aureus*- induced mastitis by targeting aryl hydrocarbon receptor

**DOI:** 10.3389/fmicb.2025.1645561

**Published:** 2025-10-15

**Authors:** Lihua Zhao, Lei Jin, Yunhe Fu, Bin Yang

**Affiliations:** ^1^Department of Breast Surgery, China-Japan Union Hospital of Jilin University, Changchun, Jilin, China; ^2^Department of Anesthesiology, China-Japan Union Hospital of Jilin University, Changchun, Jilin, China; ^3^Department of Clinical Veterinary Medicine, College of Veterinary Medicine, Jilin University, Changchun, Jilin, China

**Keywords:** mastitis, indole-3-propionic acid, ZO-1, inflammation, AhR

## Abstract

**Background:**

Indole-3-propionic acid (IPA) is a tryptophan metabolite produced by intestinal bacteria, which has functions such as penetrating tissue barriers and reducing tissue inflammatory reactions. In the present study, the therapeutic effect of IPA on *Staphylococcus aureus* (*S. aureus)*-induced mastitis was investigated.

**Methods:**

A mouse model of mastitis was established using breast injection of *S. aureus*. Except for the control group, all other mice were given oral administration of IPA. Hematoxylin eosin (H&E) staining was used to detect pathological changes in mouse mammary tissue. ELISA was used to detect TNF-α and IL-1β levels. Western blot was used to detect protein expression.

**Results:**

As the results demonstrated, IPA treatment obviously attenuated *S. aureus*-induced mammary pathological injury, myeloperoxidase (MPO) activity, malondialdehyde (MDA) content, TNF-α, and IL-1β levels. Meanwhile, IPA treatment could restore blood-milk barrier, as confirmed by up-regulating the expression of ZO-1 and occludin. *In vitro*, IPA could inhibit TNF-α and IL-1β production and the activation of NF-κB and NLRP3 induced by *S. aureus*. Furthermore, IPA could increase the expression of aryl hydrocarbon receptor (AhR). In addition, the inhibition of IPA on *S. aureus*-induced inflammation was reversed by AhR inhibitor.

**Conclusion:**

In conclusion, the results suggested that IPA inhibited *S. aureus*-induced mastitis through inhibition inflammation and restoring blood-milk barrier by activating AhR.

## 1 Introduction

Mastitis is an inflammatory disease that occurs in lactating women (Spencer, 2008). Patients may have residual lumps in the breast or ducts, which can manifest as breast pain and seriously affect their physical and mental health (Nair et al., 2015). At present, the pathogenesis of mastitis is not very clear, and most studies believe that it is related to inflammation and immune dysfunction in the body (Ingman et al., 2014). Numerous factors can induce mastitis, among which pathogen infection is considered the main factor leading to mastitis (Sordillo, 2005). *S. aureus* is one of the most important bacteria that causes mastitis (Jurado et al., 2025). It primarily affects lactating women (puerperal mastitis), with significant impacts on human health. *S. aureus* invades the mammary gland through the nipple orifice and adheres to mammary epithelial cells (Li et al., 2022). It then multiplies rapidly and secretes virulence factors (e.g., hemolysins, enterotoxins, coagulase) that damage mammary tissue, trigger an immune response and cause local inflammation (Le Maréchal et al., 2011). Western medicine often uses antibiotics or surgical methods for mastitis treatment (Yang et al., 2019). Currently, the severe situation of antibiotic resistance has certain limitations in antibiotic treatment plans (Li X. P. et al., 2023). Anti-inflammatory medications are often used alongside antibiotics to alleviate pain and reduce swelling. Non-steroidal anti-inflammatory drugs (NSAIDs) like ibuprofen are preferred in humans for lactational mastitis, as they are generally safe for breastfeeding (Krömker et al., 2021). In animals, NSAIDs such as flunixin meglumine help manage discomfort and systemic inflammation, though prolonged use may have gastrointestinal side effects (Li X. P. et al., 2023). Therefore, it is crucial to find safe and effective formulations to prevent and treat mastitis. From current research and practice, natural metabolites are a promising direction.

Indole-3-propionic acid is a gut-derived tryptophan metabolite, which has inhibitory effects on inflammation and oxidative stress, and regulates metabolism (Chen et al., 2024; Konopelski and Mogilnicka, 2022). IPA and other natural metabolites stand out as superior options for managing cow mastitis due to their unique mechanisms of action, safety profiles, and alignment with sustainable dairy farming practices. By combining targeted anti-inflammatory, antimicrobial, and tissue-supporting properties with minimal risk of resistance and residue, they address the limitations of antibiotics and conventional drugs. For dairy farmers, this translates to healthier cows, higher-quality milk, and compliance with evolving sustainability standards-making them a superior long-term solution for combating mastitis. IPA can enhance intestinal barrier function and alter the composition of intestinal microbiota (Li et al., 2021). IPA could attenuate atherosclerosis by promoting reverse cholesterol transport (Xue et al., 2022). Also, IPA obviously attenuated inflammatory response in osteoarthritis rat model and in IL-1β stimulated chondrocytes (Zhuang et al., 2023). A recent study showed that IPA could inhibit liver injury induced by sepsis in mice (Wang et al., 2023). In addition, IPA could protect blood-brain barrier in neonatal rats through inhibiting inflammation and increasing junction protein expression (Zhao et al., 2022c). However, the therapeutic role and mechanism of IPA on mastitis has not been investigated. Therefore, in the present study, we aimed to investigated the therapeutic role of IPA on *S. aureus*-induced mastitis *in vivo*.

## 2 Materials and methods

### 2.1 Reagents

Indole-3-propionic acid was obtained from Aladdin (#I184250). TNF-α (#430901) and IL-1β (#432615) assay kits were purchased from Biolegend (CA, United States). The antibodies for IκBα (#4812), p-IκBα (#9246) NF-κB p65 (#8242), NF-κB p-p65 (#3033), NLRP3 (#15101), ZO-1 (#5406), Occludin (#91131), AhR (#83200), and β-actin (4,967) were purchased from CST (CA, United States).

### 2.2 Experimental design and grouping

Sixty female C57BL/6J lactating mice were purchased from Jilin University. All the animal experiments were approved by the Institutional Animal Care and Use Committee of Jilin University. The mice were raised in a constant temperature and ventilated environment, with free diet and water intake. After 1 week of adaptive feeding, the experiment began. Sixty mice were grouped five groups and each group contain 12 mice: control group, *S. aureus* group, *S. aureus* + IPA (5, 10, 20 mg/kg) groups. *S. aureus*-induced mastitis model was established as previously reported (Bao et al., 2023). IPA (5, 10, 20 mg/kg) was given orally for 5 days before the model induced. The doses of IPA used in this study was based on previous studies (Hwang et al., 2009; Wang et al., 2023). The mice of the control group were given the same amount of solvent. 24 h after *S. aureus* treatment, the mammary tissues were collected for subsequently experiment.

### 2.3 H&E staining

After 24 h of fixation in a 4% paraformaldehyde solution, mouse breast tissue was embedded in paraffin and sliced, with a thickness of approximately 5 μm. After H&E staining, pathological changes in breast tissue were observed under an optical microscope (Olympus, Tokyo, Japan) (200 × magnification). The mammary pathological scores were calculated as previously described (Zhao et al., 2023).

### 2.4 ELISA assay

The mammary tissues were collected, homogenized on ice, and centrifuged at 3,000 r/min for 10 min. Then, the homogenates were collected and TNF-α and IL-1β levels in the homogenates were tested by the ELISA kits (Zhao et al., 2022b).

### 2.5 Immunohistochemistry

The paraffin sections of breast tissue were sequentially subjected to dewaxing, hydration, antigen repair, and inhibition of endogenous peroxidase. The primary antibody was incubated overnight at 4 °C, while the secondary antibody was incubated at room temperature for 30 min. The steps included color development, re staining, dehydration, transparency, and sealing. Subsequently, the samples were observed and photographed under a microscope (Zhao et al., 2022a).

### 2.6 Western blot analysis

Total protein was extracted from mouse breast tissue using RIPA buffer, and the protein content was determined using the BCA protein quantification kit. The protein samples (30 μg) were separated via 12% SDS-PAGE gel electrophoresis and the membrane was transferred to PVDF membrane. Then, the membrane was blocked with 5% skim milk for 4 h and then incubated with primary antibodies at 4 °C overnight. After three washes with TBS-T, the membrane was incubated with secondary antibody at room temperature for 2 h. Finally, the proteins were visualized using an enhanced chemiluminescence solution. ImageJ software was used to analyze the grayscale values of the target protein and internal reference protein. β-actin was used as an internal reference protein in this experiment (Zhao et al., 2023).

### 2.7 *In vitro* study

Mouse mammary epithelial cells (MMECs) (ATCC, CRL-3062) were purchased from ATCC and cultured in DMEM containing 10% FBS at 37 °C and 5% CO_2_, and the fresh culture medium were changed every 24 h. The effect of IPA on the cell viability was tested by MTT assay. The cells were incubated with IPA and stimulated by *S. aureus* (10^5^ CFU). The gene expression of TNF-α and IL-1β were tested by qRT-PCR. The primers were based on previous studies (Li K. F. et al., 2023). For AhR inhibitory experiment, CH-2232191 (10 μM) was given 2 h before *S. aureus* and IPA treatment.

### 2.8 qRT-PCR

Total RNA was extracted from MMECs using TRIzol reagent (Invitrogen, Carlsbad, CA, United States) following the manufacturer’s instructions. Quantitative real-time PCR (qRT-PCR) was conducted on a StepOnePlus™ Real-Time PCR System (Thermo Fisher Scientific) using TB Green^®^ Premix Ex Taq II (Takara Bio Inc.). The 20 μL reaction system contained 10 μL TB Green Premix Ex Taq II, 0.4 μL forward primer (10 μM), 0.4 μL reverse primer (10 μM), 2 μL cDNA template (1:5 dilution), and 7.2 μL RNase-free water. The thermal cycling conditions were: initial denaturation at 95 °C for 30 s, followed by 40 cycles of denaturation at 95 °C for 5 s and annealing/extension at 60 °C for 30 s. β-actin served as the reference gene. Relative TNF-α and IL-1β expression was calculated via 2^–ΔΔCt^ method. All samples had triplicate technical repeats and three biological replicates. Primers were synthesized by Sangon Biotech.

### 2.9 Statistical analysis

Statistical analysis was conducted using SPSS 23.0 software. The measurement data is expressed as mean ± standard deviation. Multiple inter group data comparisons were conducted using one-way analysis of variance, and graphs were created using GraphPad Prism 8 software. *P* < 0.05 indicates that the difference is statistically significant.

## 3 Results

### 3.1 Effects of IPA on MMECs viability and inflammatory cytokines expression

**FIGURE 1:**
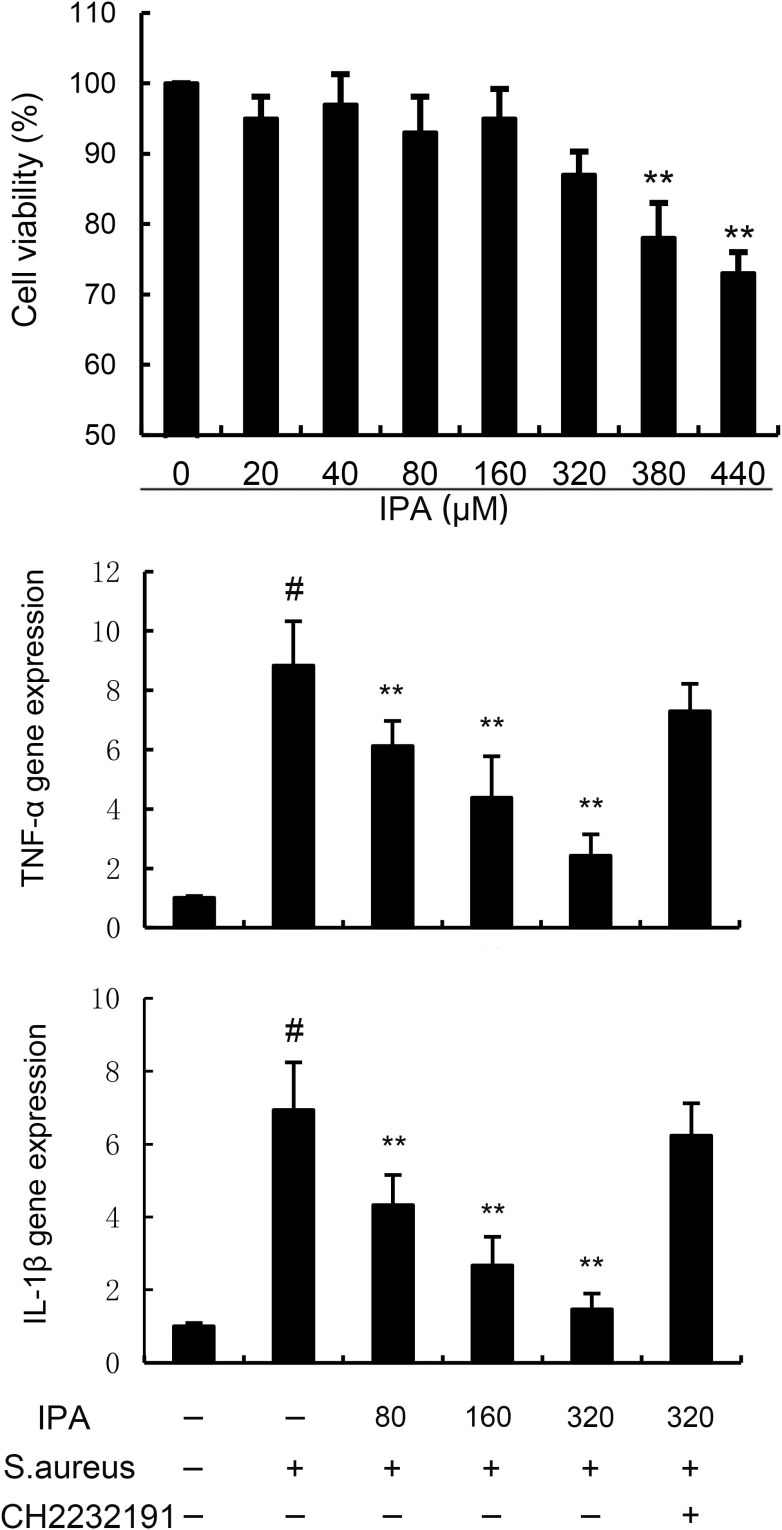
Effects of indole-3-propionic acid (IPA) on mouse mammary epithelial cells (MMECs) viability and inflammatory cytokines expression. The values presented are the mean ± SD (*n* = 6). ^#^*P* < 0.01 is significantly different from control group; **P < 0.01 are significantly different from *S. aureus* group.

To investigate whether IPA has toxic effects on MMECs, MTT assay was used to detect the effect of different concentrations of IPA on MMECs cell viability. According to Figure 1, IPA at concentrations of 20, 40, 80, 160, 320 μM had no significant effect on the viability of MMECs (*P* > 0.05), while IPA at concentrations of 380 and 440 μM significantly reduced the viability of MMECs compared to the control group (*P* < 0.05). Meanwhile, TNF-α and IL-1β gene expression in *S. aureus*-treated cells were higher than the control. In addition, IPA treatment alleviated the expression of TNF-α and IL-1β, which were increased by *S. aureus* stimulation (Figure 1).

### 3.2 IPA inhibits *S. aureus*-induced NF-κB activation in MMECs

**FIGURE 2:**
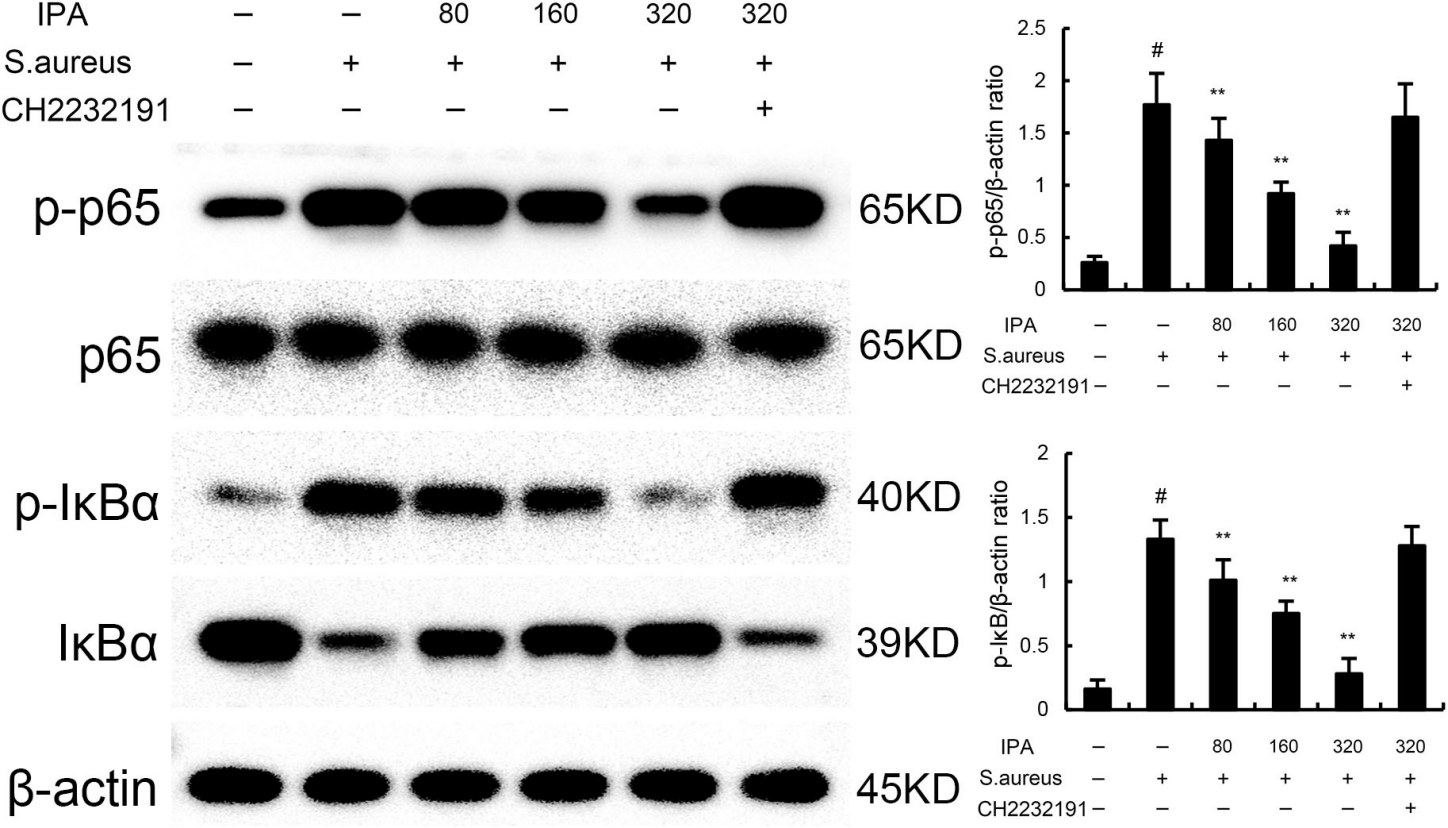
Effect of IAld on NF-κB activation in mouse mammary epithelial cells (MMECs). The values presented are the mean ± SD (*n* = 3). ^#^*P* < 0.01 is significantly different from control group; **P < 0.01 are significantly different from *S. aureus* group.

NF-κB was tested to assess the anti-inflammatory mechanism of IPA. The data indicated that the expression of phosphorylated IκBα and NF-κB p65 in *S. aureus*-treated cells were higher than the control mice. In addition, IPA treatment attenuated the expression of phosphorylated IκBα and NF-κB p65, which were increased by *S. aureus* stimulation (Figure 2 and Supplementary material).

### 3.3 IPA inhibits *S. aureus*-induced NLRP3 activation in MMECs

**FIGURE 3:**
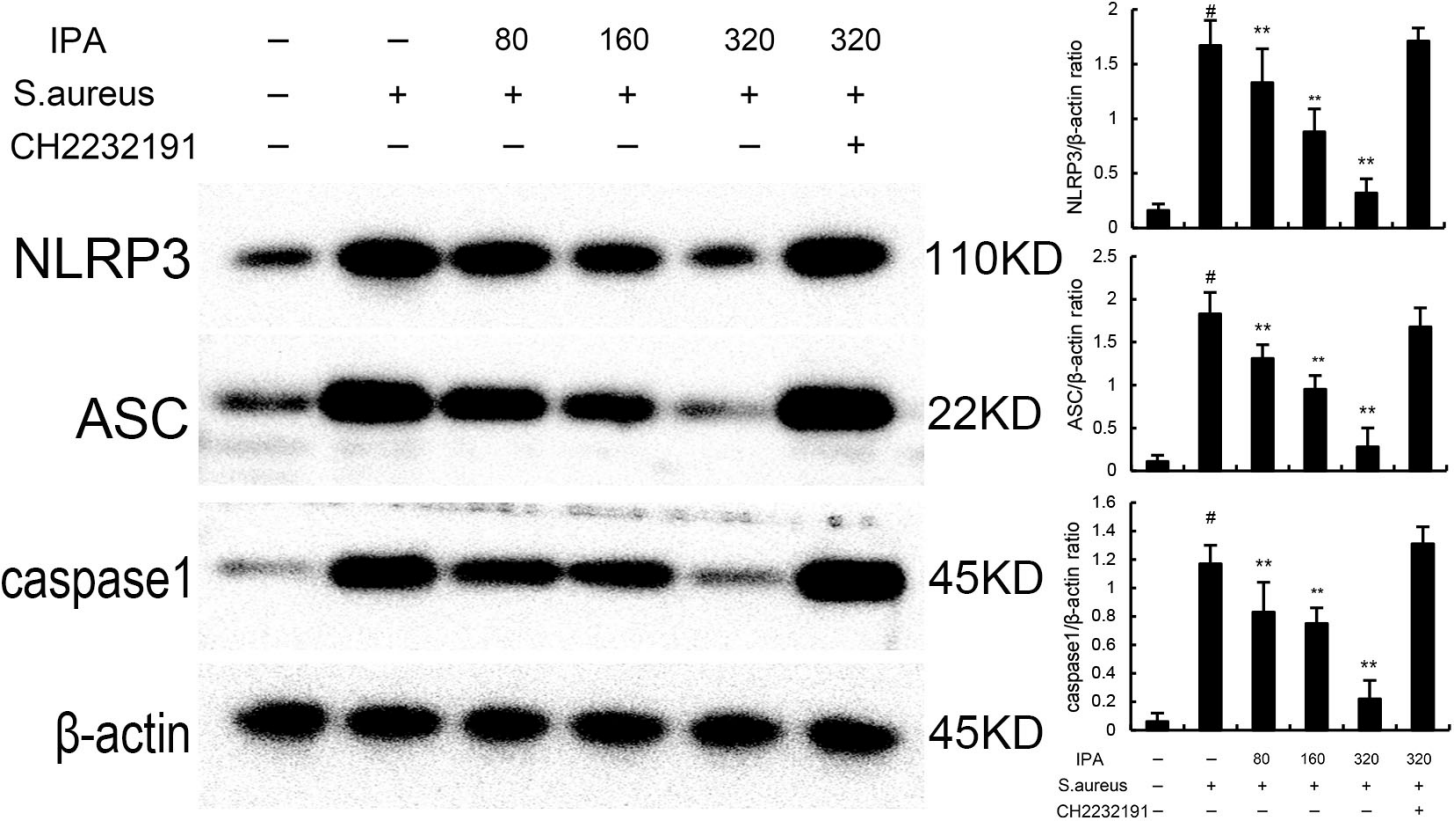
Effect of IAld on Nod like receptor protein 3 (NLRP3) activation in mouse mammary epithelial cells (MMECs). The values presented are the mean ± SD (*n* = 3). ^#^*P* < 0.01 is significantly different from control group; **P < 0.01 are significantly different from *S. aureus* group.

Nod like receptor protein 3 inflammasome was tested to assess the anti-inflammatory mechanism of IPA. The data indicated that the expression of NLRP3, ASC, and caspase1 in *S. aureus*-treated cells were higher than the control mice. In addition, IPA treatment attenuated the expression of NLRP3, ASC, and caspase1, which were increased by *S. aureus* stimulation (Figure 3).

### 3.4 IPA inhibits *S. aureus*-induced inflammation through activating AhR

**FIGURE 4:**
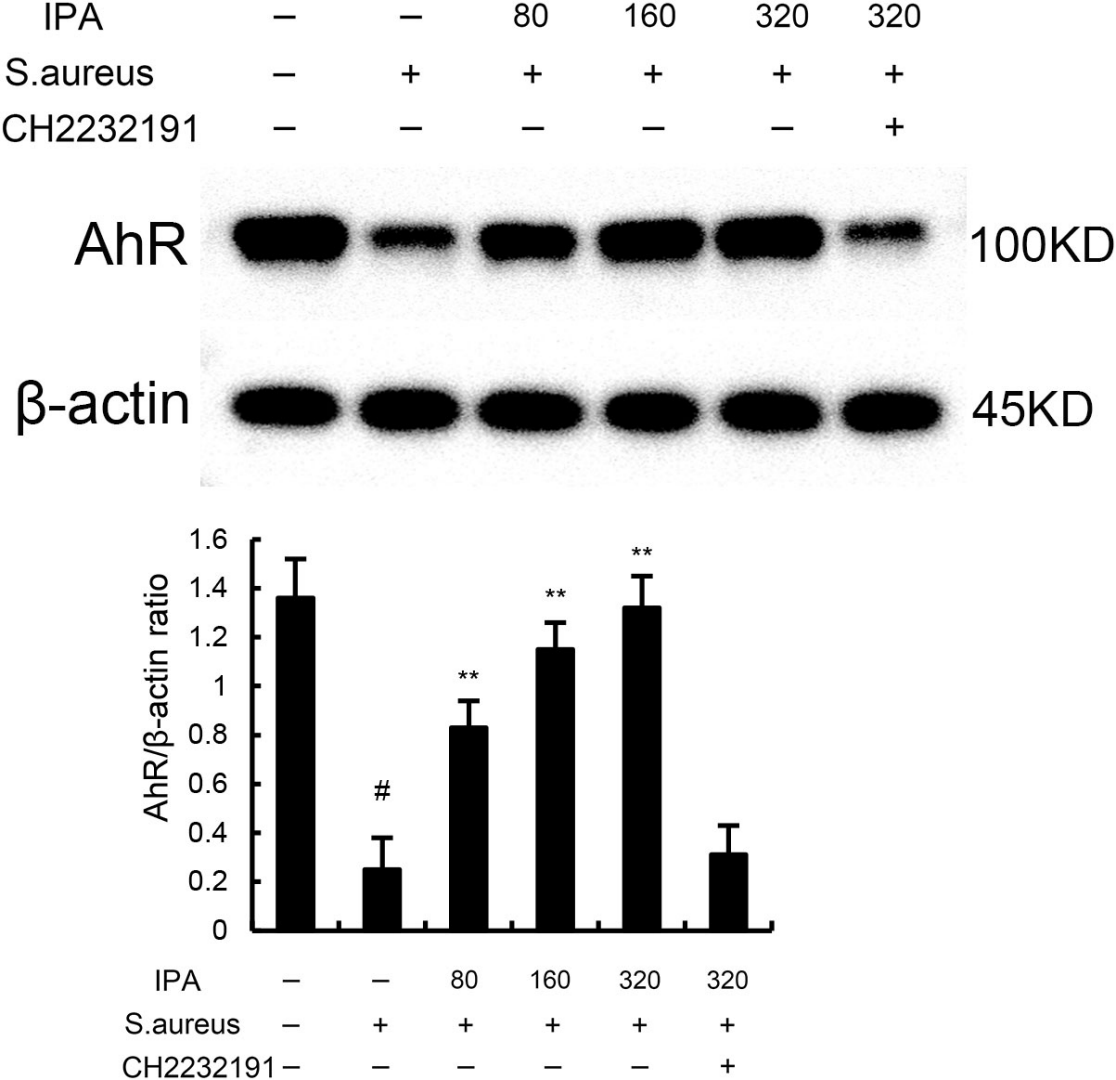
The protective role of IAld on *S. aureus*-induced mastitis is depended on aryl hydrocarbon receptor (AhR). The values presented are the mean ± SD (*n* = 3). #*P* < 0.01 is significantly different from control group; **P < 0.01 are significantly different from *S. aureus* group.

The effects of IPA on AhR expression were measured by western blot. The data indicated that IPA treatment increased the expression of AhR in a dose-dependent manner (Figure 4). Meanwhile, the inhibition of IPA on *S. aureus*-induced TNF-α and IL-1β expression, NF-κB, and NLRP3 activation were inhibited by AhR inhibitor CH-2232191 (Figures 1–3).

### 3.5 Effects of IPA on *S. aureus*-induced mammary pathological injury

**FIGURE 5:**
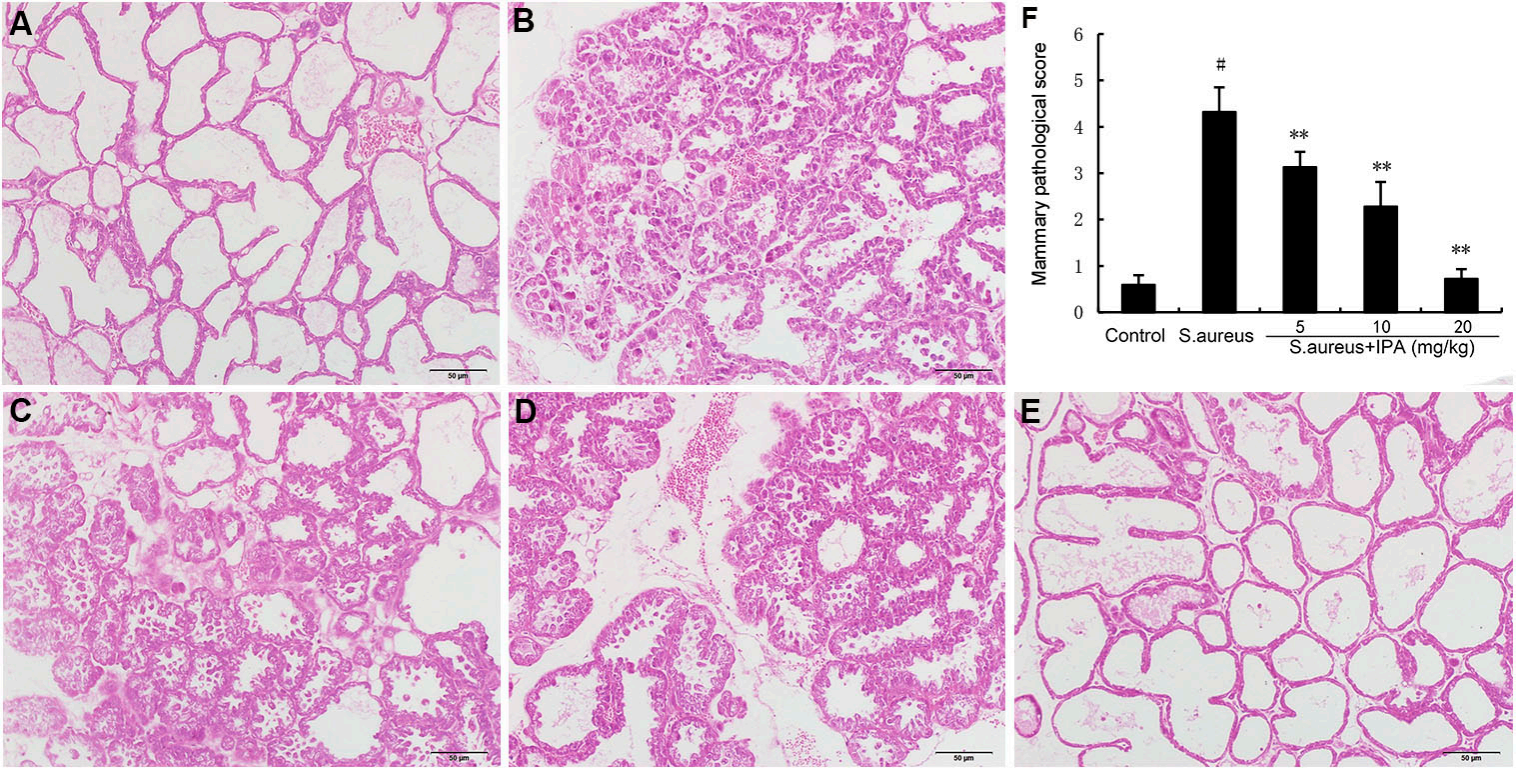
Effects of indole-3-propionic acid (IPA) on *S. aureus*-induced mammary histopathological changes. Histopathologic sections of mammary tissues (H&E, × 100). **(A)** Control, **(B)** *S. aureus* group, **(C–E)** Control group, *S. aureus* + IPA (5, 10, 20 mg/kg) groups, **(F)** The mammary pathological score. ^#^*P* < 0.01 is significantly different from control group; **P < 0.01 are significantly different from *S. aureus* group.

The mammary gland tissue acini of the model group mice collapsed, and there was a large amount of inflammatory infiltration in the cavity. IPA treatment significantly attenuated *S. aureus*-induced mammary injury (Figure 5).

### 3.6 Effects of IPA on MPO activity and MDA level

**FIGURE 6:**
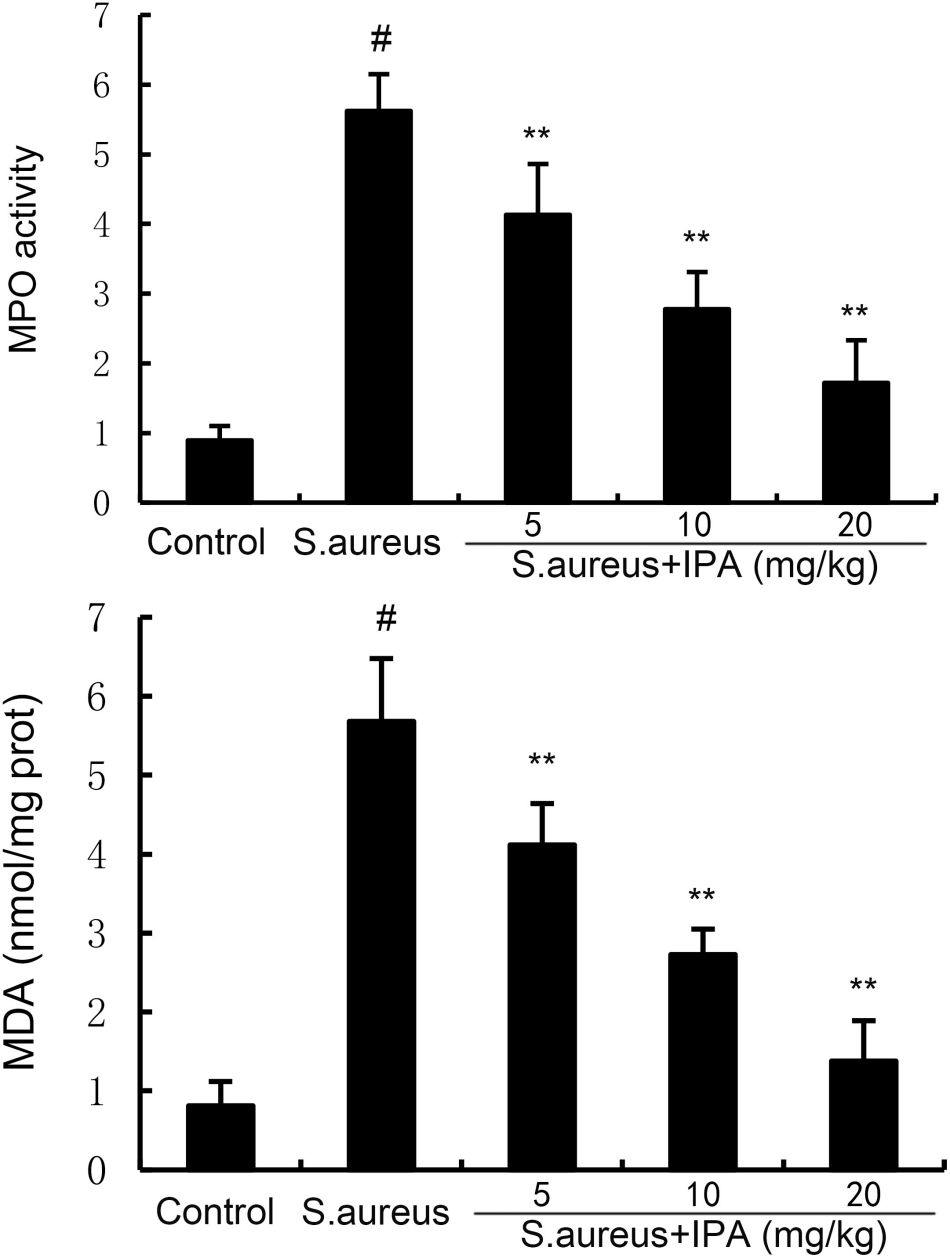
Effect of indole-3-propionic acid (IPA) on myeloperoxidase (MPO) activity and malondialdehyde (MDA) content in mammary gland. The values presented are the mean ± SD (*n* = 6). ^#^*P* < 0.01 is significantly different from control group; **P < 0.01 are significantly different from *S. aureus* group.

Myeloperoxidase is primarily expressed in neutrophils and is released during neutrophil activation. In mastitis, bacterial infection triggers the recruitment of neutrophils to the mammary gland. Therefore, we assessed the recruitment of neutrophils to the mammary gland by detecting MPO activity. MDA is a major end-product of lipid peroxidation and a biomarker of oxidative stress. Therefore, we assessed the anti-oxidative role of IPA by detecting MDA level. The data indicated that the MPO and MDA contents in mammary tissues of *S. aureus*-treated mice were higher than the control mice. In addition, IPA treatment alleviated the contents of MPO and MDA, which were increased by *S. aureus* stimulation (Figure 6).

### 3.7 IPA alleviates *S. aureus*-induced TNF-α and IL-1β production

**FIGURE 7:**
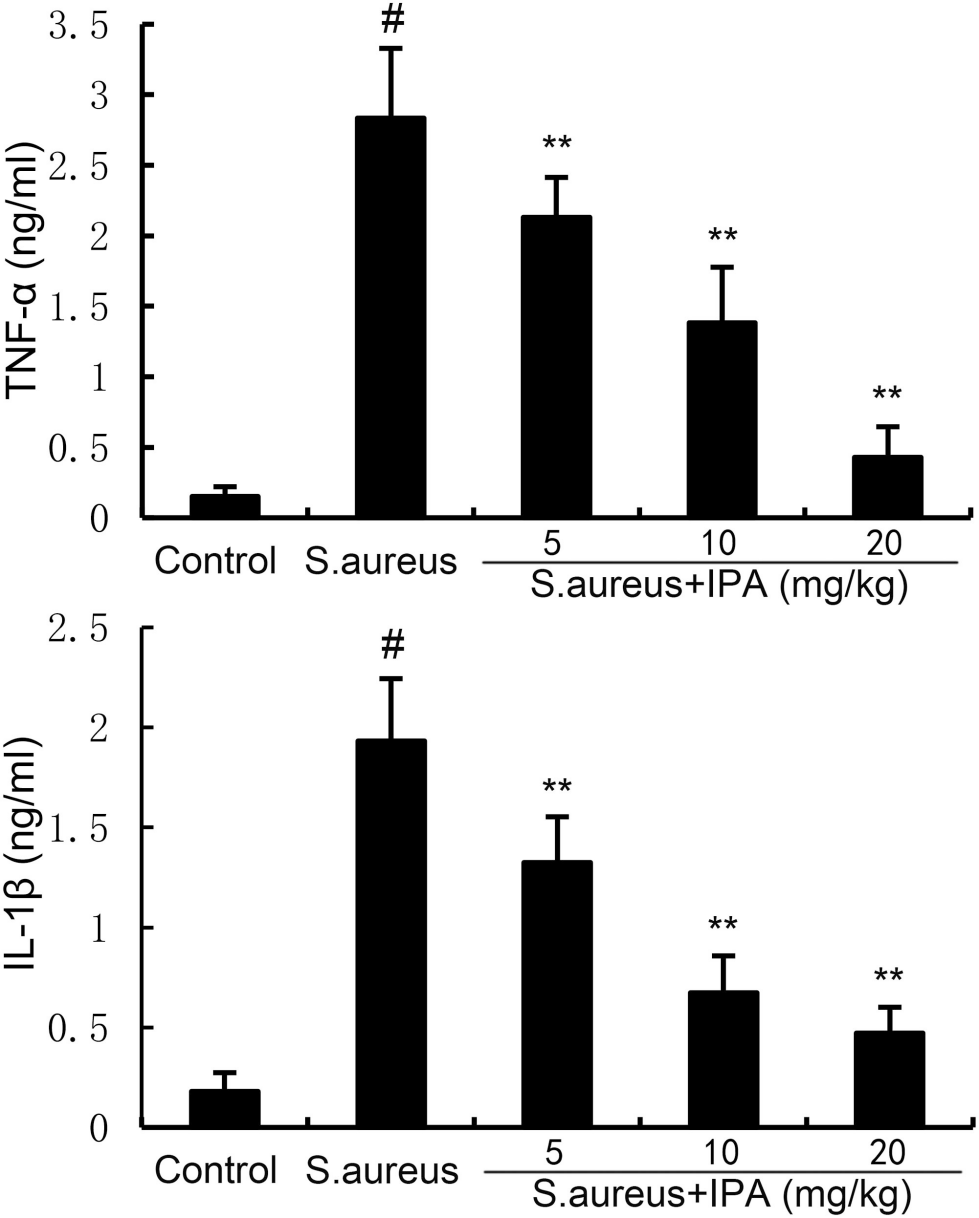
Effect of indole-3-propionic acid (IPA) on inflammatory cytokine production in mammary gland. The values presented are the mean ± SD (*n* = 6). ^#^*P* < 0.01 is significantly different from control group; **P < 0.01 are significantly different from *S. aureus* group.

TNF-α and IL-1β levels were measured by ELISA method. The data indicated that TNF-α and IL-1β levels in mammary tissues of *S. aureus*-treated mice were higher than the control mice. In addition, IPA treatment alleviated the contents of TNF-α and IL-1β, which were increased by *S. aureus* stimulation (Figure 7).

### 3.8 IPA alleviates *S. aureus*-induced blood-milk barrier injury

**FIGURE 8:**
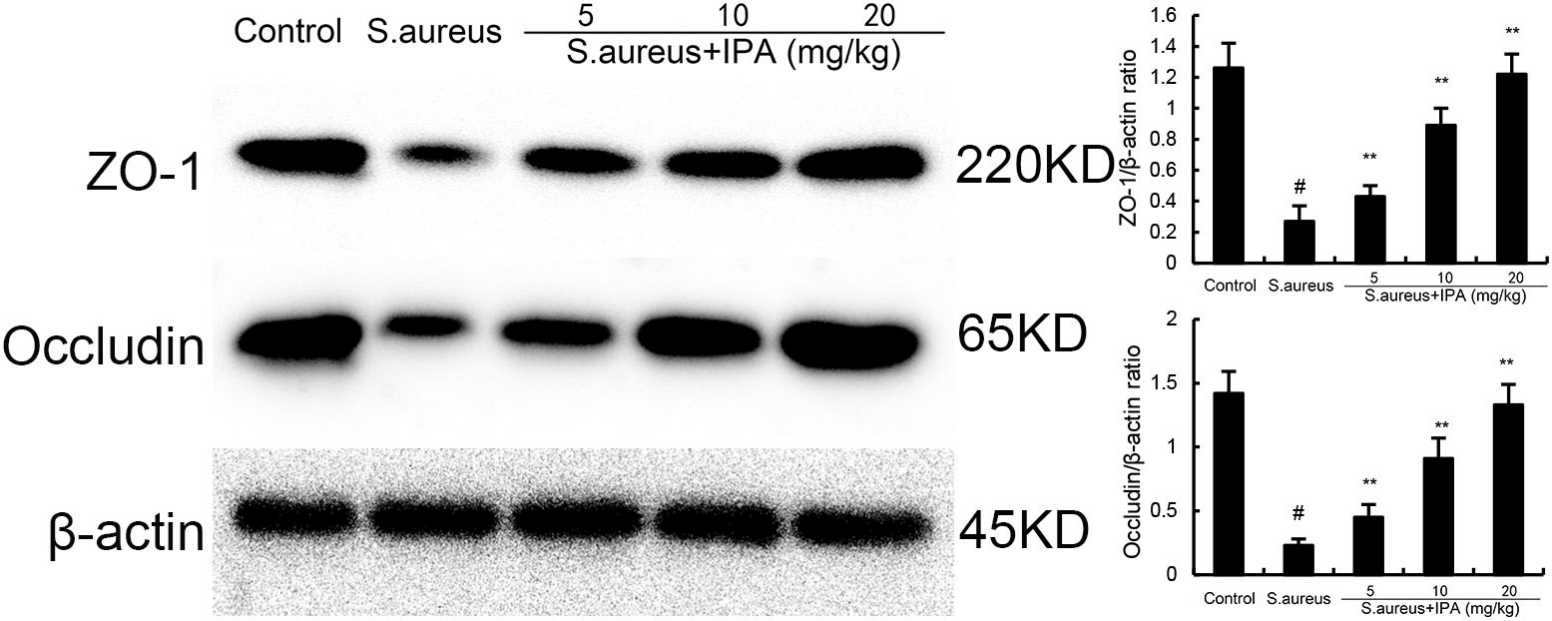
Effect of indole-3-propionic acid (IPA) on ZO-1 and occludin expression in mammary gland. The values presented are the mean ± SD (*n* = 3). ^#^*P* < 0.01 is significantly different from control group; **P < 0.01 are significantly different from *S. aureus* group.

ZO-1 and occludin were tested to assess the effects of IPA on blood-milk barrier. The data indicated that ZO-1 and occludin expression in mammary tissues of *S. aureus*-treated mice were lower than the control mice. In addition, IPA treatment up-regulated the expression of ZO-1 and occludin, which were decreased by *S. aureus* stimulation (Figure 8).

## 4 Discussion

In recent years, the clinical management of *S. aureus*-induced mastitis in lactating women has faced persistent challenges. Traditional antibiotic therapy, while widely used, is increasingly limited by the emergence of drug-resistant strains (e.g., methicillin-resistant *S. aureus*, MRSA) and concerns over antibiotic residues in milk, driving the urgent need to explore green, safe, and mechanism-based alternative therapies. IPA is an intestinal metabolite of tryptophan, produced by the breakdown of tryptophan by gut microbiota such as *Lactobacillus reuterii*, *Akkermann’s* bacteria, and *Clostridium* genus (Hou et al., 2023). At present, a large number of studies have shown that IPA has the effects of inhibiting inflammation, antioxidant stress, neuroprotection, and inhibiting the risk factors of cardiovascular and cerebrovascular diseases, such as hypertension, diabetes, obesity, etc., (Konopelski et al., 2021; Zhang et al., 2022). Building on these findings, our study aimed to test the hypothesis that IPA could exert therapeutic effects on *S. aureus*-induced mastitis. The data showed IPA could inhibit *S. aureus*-induced mastitis through inhibiting inflammation and restoring blood-milk barrier. The results not only validate this hypothesis but also reveal a novel mechanistic axis (IPA-AhR) that bridges gut metabolites to mammary gland homeostasis, offering unique insights into mastitis pathogenesis and treatment.

The integrity of the blood milk barrier is a prerequisite for ensuring the normal lactation function of breast tissue, and tight connections are the key structures to maintain the integrity of the blood milk barrier (Wellnitz and Bruckmaier, 2021). Research has found that the integrity of the blood milk barrier is disrupted after mastitis, and its dysfunction leads to excessive entry of neutrophils into breast tissue, causing damage (Li K. F. et al., 2023). Therefore, maintaining the integrity of the blood milk barrier is of great significance for attenuating inflammatory damage to the mammary gland (Wall et al., 2016). Our study demonstrates, for the first time, that IPA administration reverses *S. aureus*-induced blood-milk barrier damage by upregulating the expression of ZO-1 and occludin. The results address a critical unmet need in mastitis therapy: while existing strategies often focus on suppressing inflammation after barrier disruption, IPA acts upstream to preserve or restore barrier integrity, thereby limiting the initial influx of inflammatory cells and preventing the damage.

After being infected with *S. aureus* in the breast, neutrophils in the blood will cross the blood milk barrier and enter the breast (Sordillo and Streicher, 2002). A hallmark of its pathogenicity lies in its ability to trigger oxidative stress and inflammation in host cells, which interact as a self-amplifying loop to drive tissue damage, impair immune clearance, and promote bacterial persistence (Beavers and Skaar, 2016). Under the stimulation of *S. aureus*, neutrophils will release various inflammatory cytokines TNF-α, IL-1β, and IL-6 (Akhtar et al., 2020). These inflammatory mediators can further amplify the inflammatory process and lead to mammary injury (Zhao and Lacasse, 2008). *S. aureus* secretes virulence factors and expresses cell wall components that directly or indirectly stimulate host cells to generate ROS (Cheung et al., 2021). Research has shown that some plant active ingredients can weaken *S. aureus* induced mastitis by inhibiting the production of inflammatory cytokines and oxidative stress (Fu et al., 2014). IPA has been demonstrated to be a powerful antioxidant (Hwang et al., 2009). The relationship between antioxidants and anti-inflammation is complex and intertwined. Oxidative stress and inflammation are closely linked, forming a vicious cycle that can contribute to the development and progression of many diseases (Kalacun et al., 2025). Antioxidants, including IPA, can interrupt this cycle by scavenging ROS, modulating inflammatory signaling pathways, and affecting immune cell function. In the present study, IPA treatment obviously attenuated *S. aureus*-induced TNF-α and IL-1β production. NF-κB is a transcription factor that can participate in immune and inflammatory responses (Caamaño and Hunter, 2002). The NF-κB signaling pathway promotes inflammatory factors through TNF-α and IL-1β, as well as regulating the production of chemokines such as IL-8, MCP-1, and CXCL10 to regulate the inflammatory response (Mitchell and Carmody, 2018). More and more studies have shown that inhibiting the activation of the NF-κB signaling pathway can alleviate mastitis (Khan et al., 2024). As Wang et al. found in their study, the expression of phosphorylated NF-κB p65 was significantly increased in lipopolysaccharide induced mouse mammary epithelial cells and breast tissue of mice with mastitis (Wang et al., 2017). However, after sodium butyrate treatment, the activation of the NF-κB signaling pathway was inhibited, leading to the downregulation of inflammatory cytokine IL-1β, IL-6 and TNF-α (Wang et al., 2017). When *S. aureus* invades the mammary epithelial cells, the p65 subunit of NF-κB translocates from the cytoplasm to the nucleus. After drug treatment, this process can be prevented, inhibiting NF-κB activation and achieving the effect of relieving mastitis (Jiang et al., 2017). Based on the above, NF-κB plays a crucial role in resisting inflammatory damage to breast tissue and epithelial cells. In this study, IPA significantly inhibited *S. aureus*-induced NF-κB activation.

Nod like receptor protein 3 inflammasome is an intracellular polymeric protein complex that plays an important role in inflammation and immune responses (Ahmed et al., 2024). The NLRP3 inflammasome is composed of three main components: Nod like receptor protein 3 (NLRP3), apoptosis related spot like protein (ASC), and caspase-1 (Sharma and de Alba, 2021). When the inflammasome is activated, it induces the activation of caspase-1, mediating the maturation and secretion of pro-inflammatory cytokines such as IL-1β and IL-18, and inducing cell apoptosis (Elliott and Sutterwala, 2015). It plays an important role in regulating the host’s immune response to pathogen infection and tissue repair of cell damage (Kim and Jo, 2013). When NLRP3 inflammasome is activated, it produces and releases inflammatory mediators, participating in the occurrence and development of various inflammatory diseases (Li et al., 2020). Previous study demonstrated that inhibition of NLRP3 inflammasome could attenuate *S. aureus*-induced mastitis (Yang et al., 2022). In this study, we found IPA obviously attenuated *S. aureus*-induced NLRP3 inflammasome activation. Notably, our results contrast with studies focusing on plant-derived anti-inflammatory compounds (e.g., curcumin, resveratrol) for mastitis, which often act through similar NF-κB/NLRP3 pathways but face challenges such as poor bioavailability or inconsistent gut absorption (Xu et al., 2020; Zhou et al., 2020). IPA, by virtue of being a natural gut metabolite, may overcome these limitations. It is endogenously produced by the microbiota, has proven oral bioavailability, and is well-tolerated even at high doses, making it a more translationally feasible candidate for clinical use (Jiang et al., 2023).

A central novel finding of our study is that IPA’s anti-inflammatory and barrier-protective effects are dependent on the activation of the aryl hydrocarbon receptor (AhR). AhR is a ligand-activated transcription factor traditionally linked to xenobiotic metabolism, but recent studies have established its role in regulating immune responses, epithelial barrier function, and gut-mammary crosstalk. AhR is a key regulatory factor in drug metabolism, involved in regulating cell apoptosis, proliferation, inflammatory response, and glucose and lipid metabolism, and is associated with the development of various diseases (Koutsounas et al., 2013). Previous studies demonstrated that activation AhR could inhibit NF-κB activation and inflammatory response (Deuring et al., 2019). In this study, we found IPA could increase the expression of AhR and AhR inhibitor reversed the anti-inflammatory role of IPA on *S. aureus*-induced inflamamtion, suggesting IPA protected mice against mastitis through activating AhR.

In conclusion, this paper confirms that IPA can exert a protective effect on *S. aureus*-induced mastitis through regulating the blood milk barrier and inhibiting inflammation, and preliminarily reveals that its mechanism of action is achieved by activating AhR, which subsequently inhibits NF-κB and NLRP3 signaling pathways. This finding not only identifies IPA as a promising candidate for the development of novel, safe, and effective therapies against *S. aureus*-induced mastitis but also provides new insights into targeting the gut metabolite-AhR axis to regulate mammary gland inflammation and barrier function.

## Data Availability

The original contributions presented in this study are included in this article/Supplementary material, further inquiries can be directed to the corresponding author.
