# Supplementary material for: Gut microbial metabolite indole-3-propionic acid inhibits inflammation and restores blood-milk barrier in S. aureus- induced mastitis by targeting aryl hydrocarbon receptor
Source: Front Microbiol. 2025 Oct 15;16:1645561. doi: 10.3389/fmicb.2025.1645561 (PMC12568595; doi:10.3389/fmicb.2025.1645561)

Fig 8

ZO-1 1

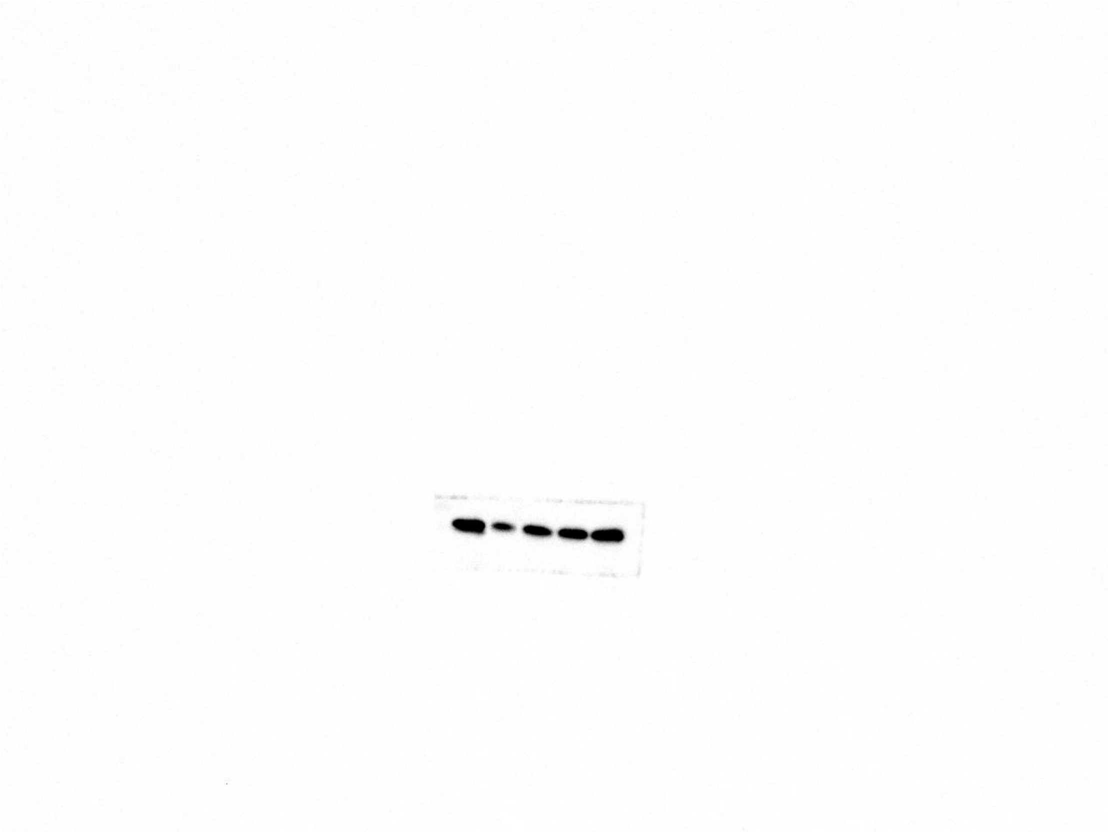

Zo-1 2

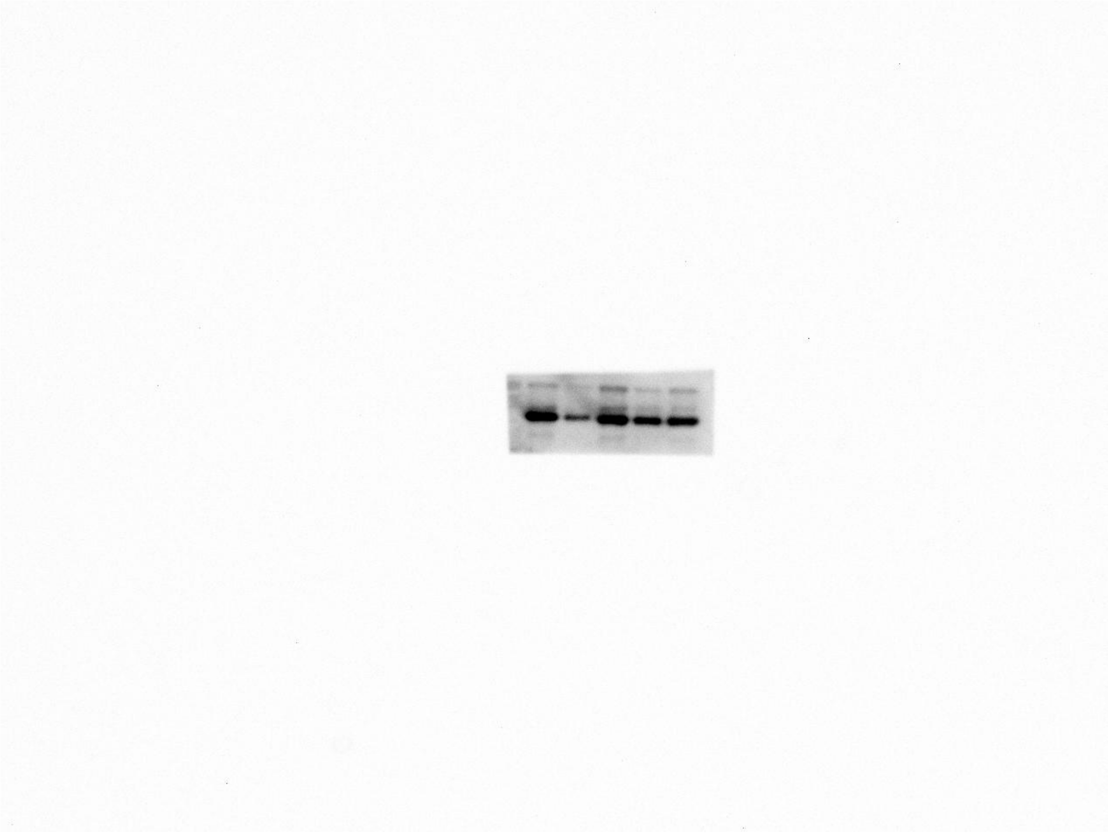

Zo-1 3

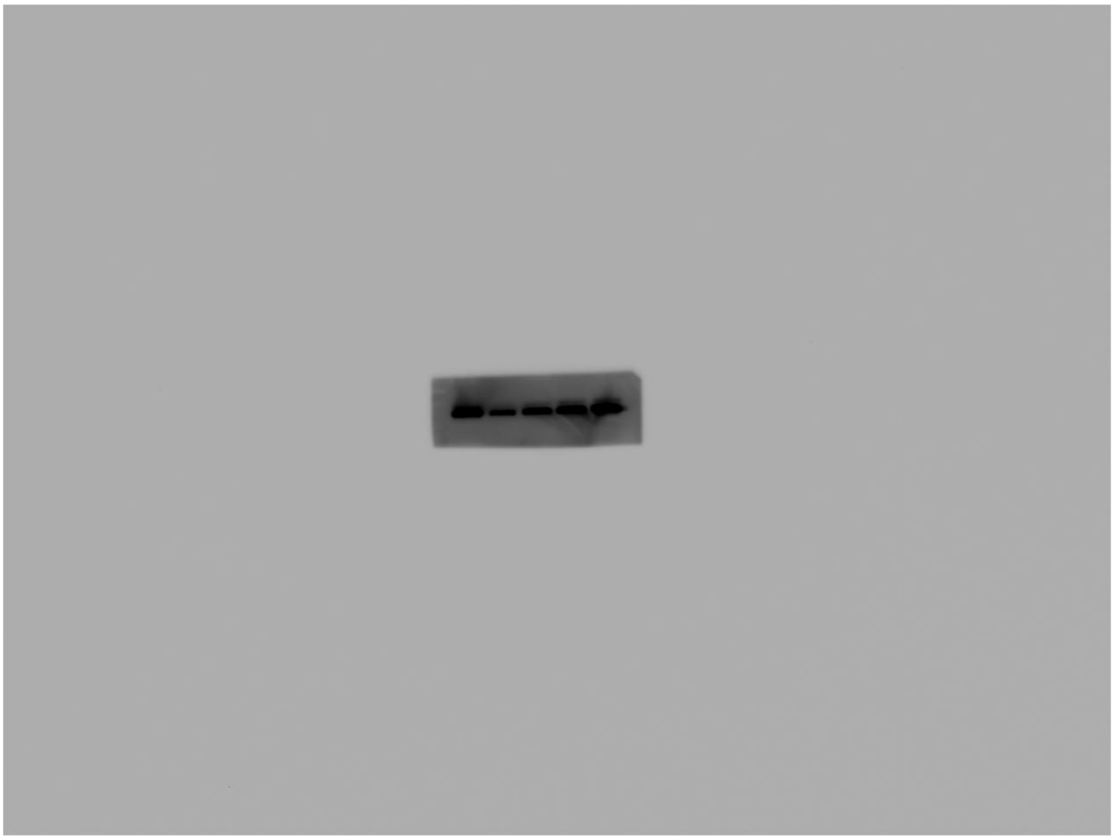

Occludin 1

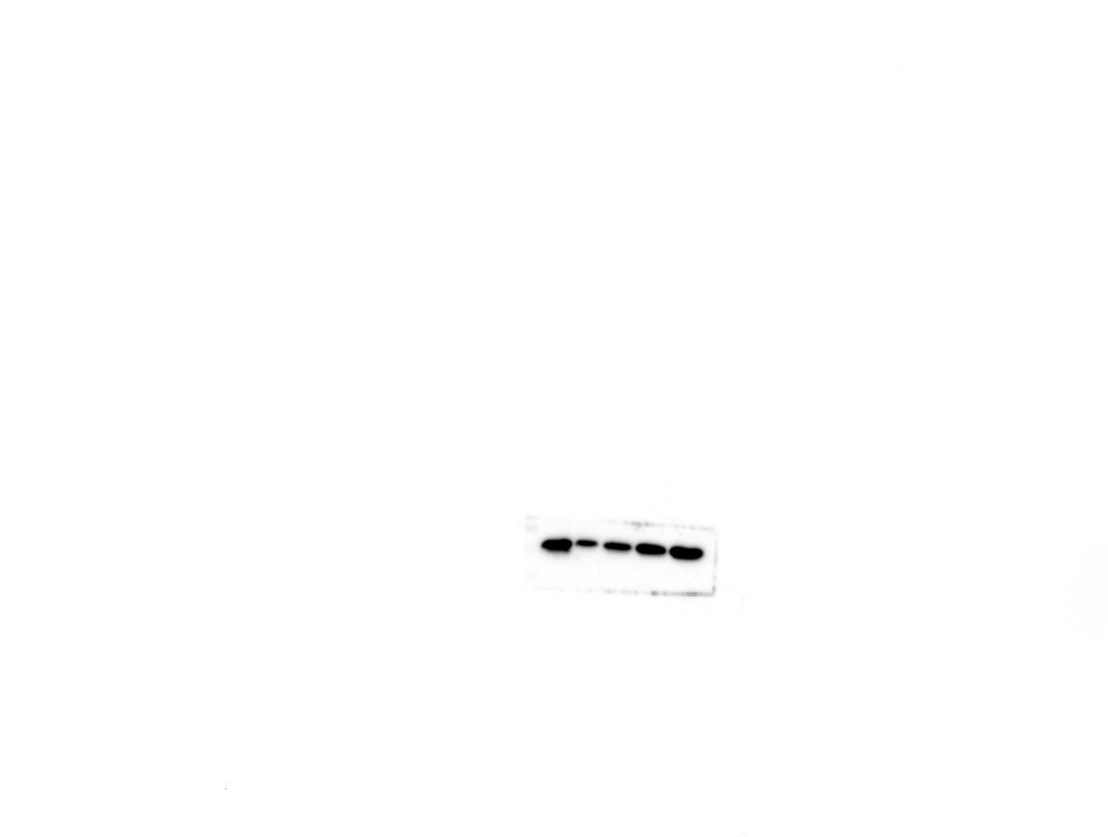

Occludin 2

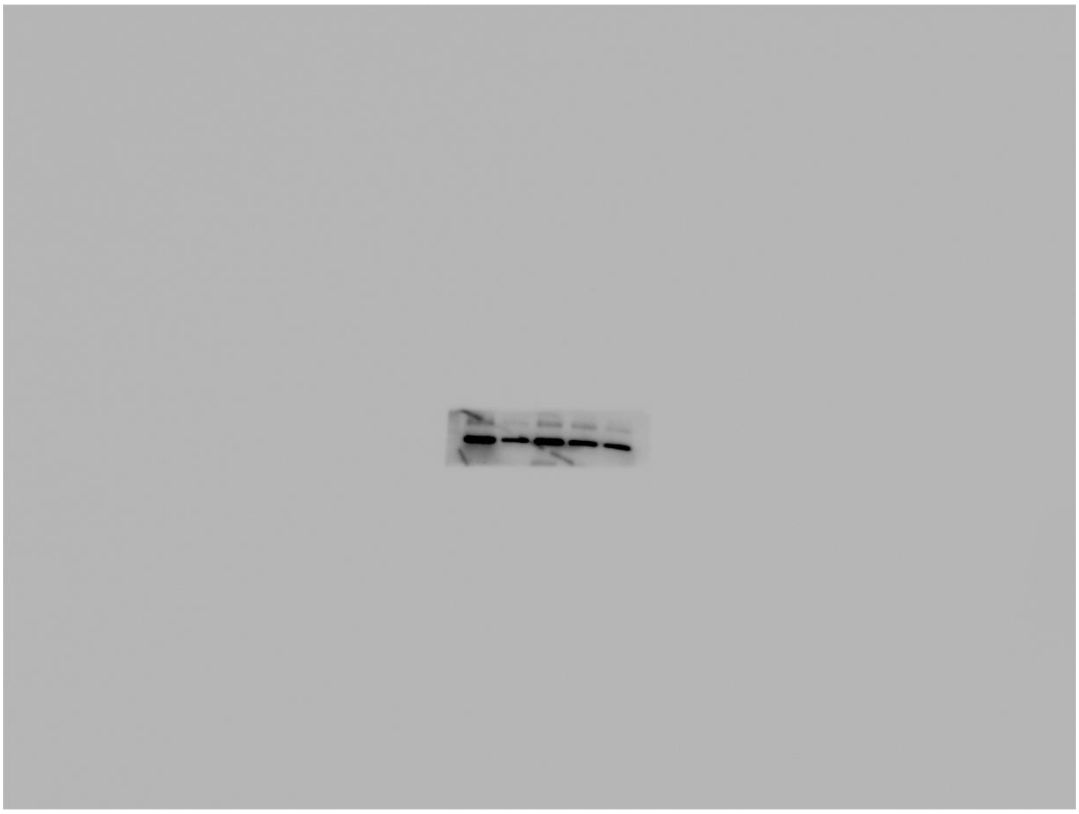

Occludin 3

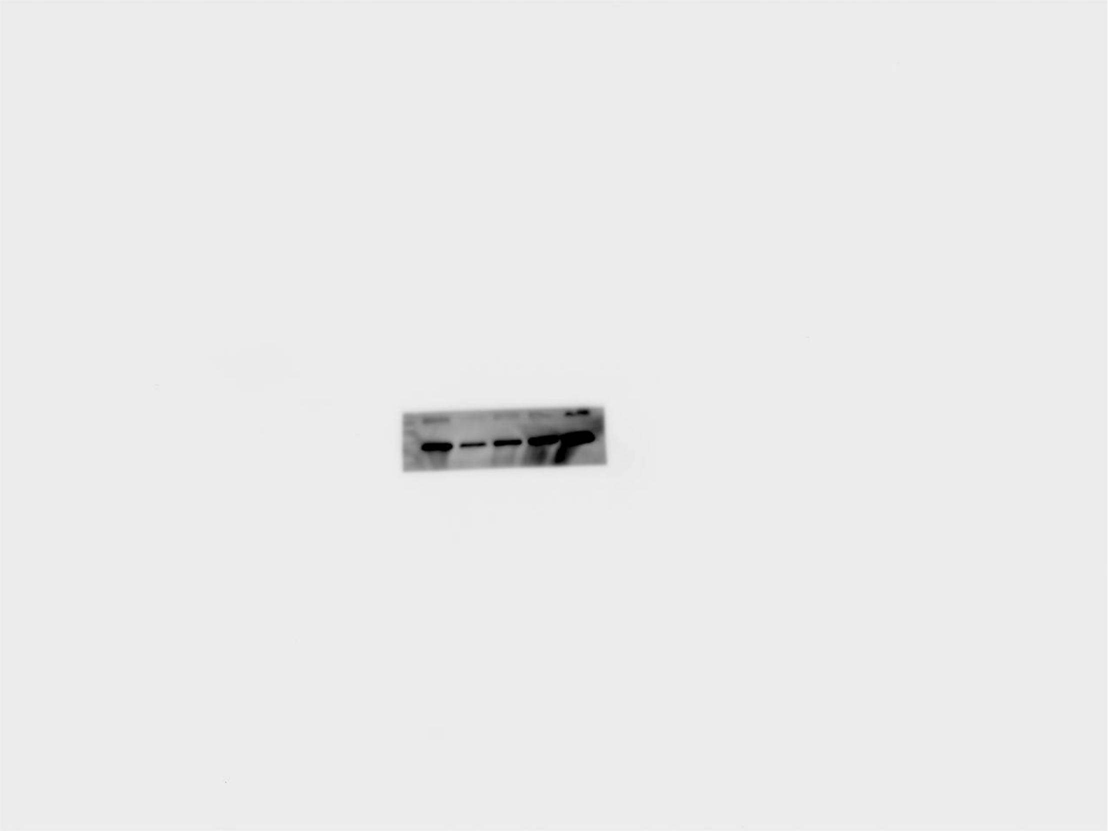

Actin 1

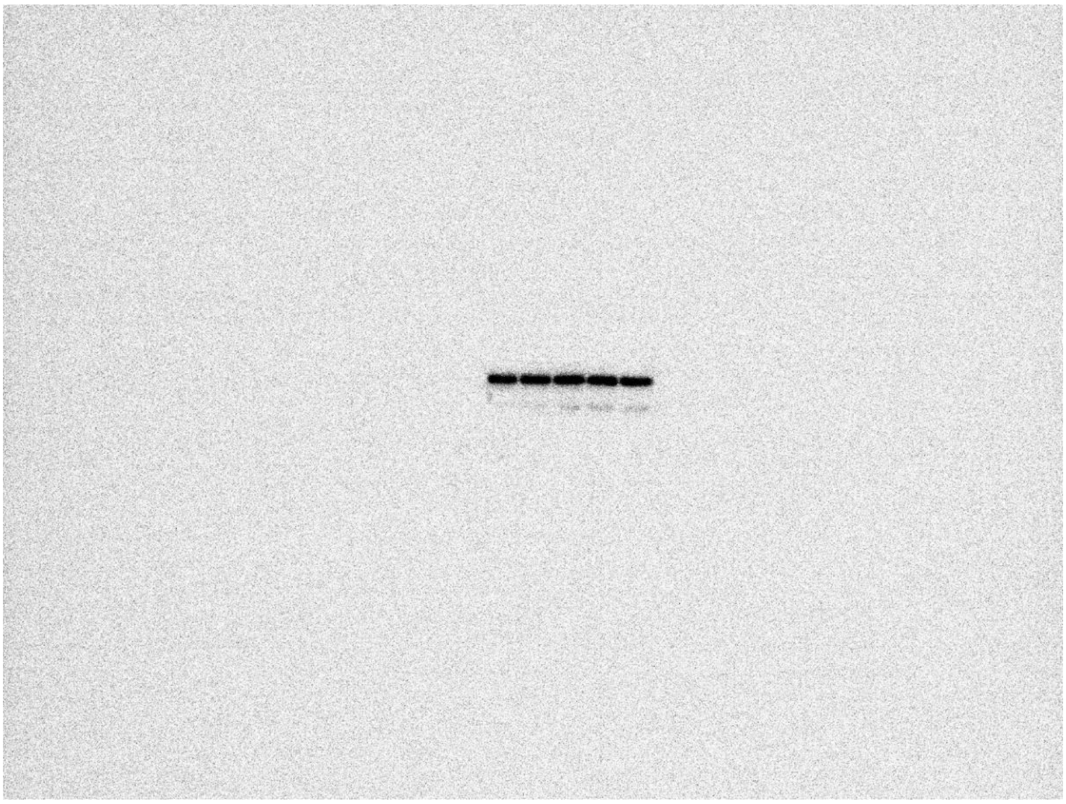

Actin 2

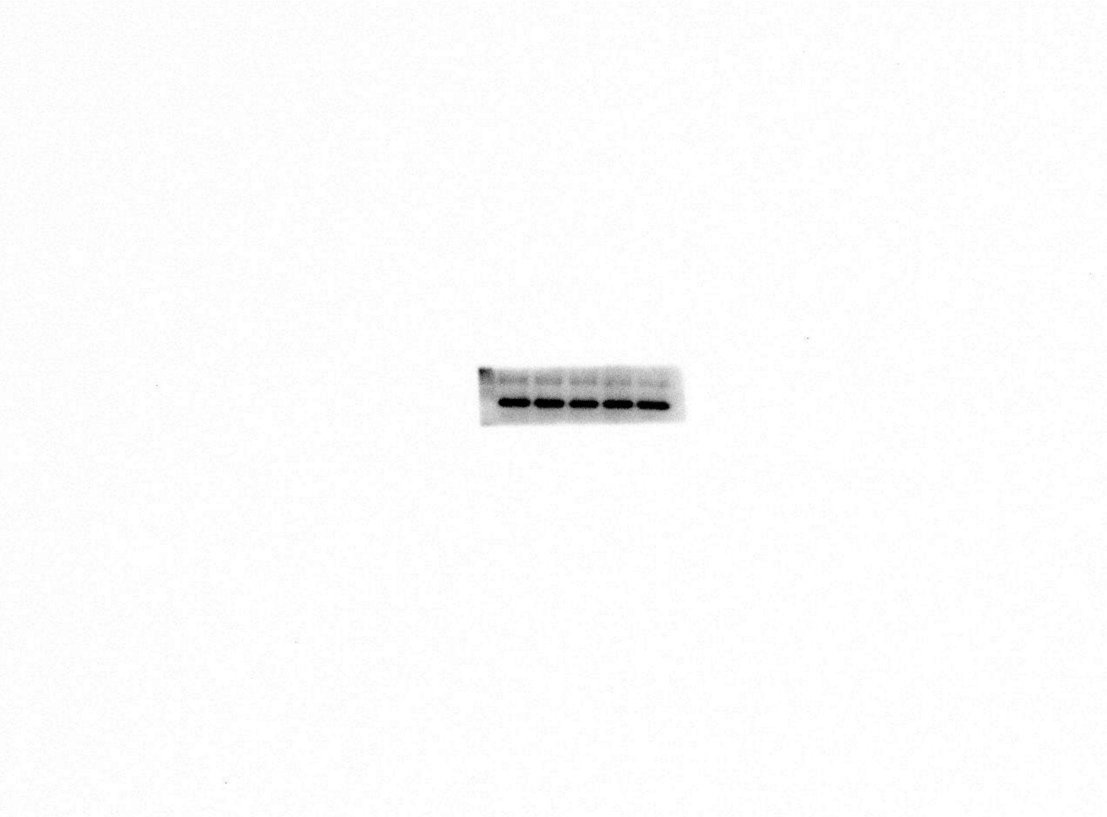

Actin 3

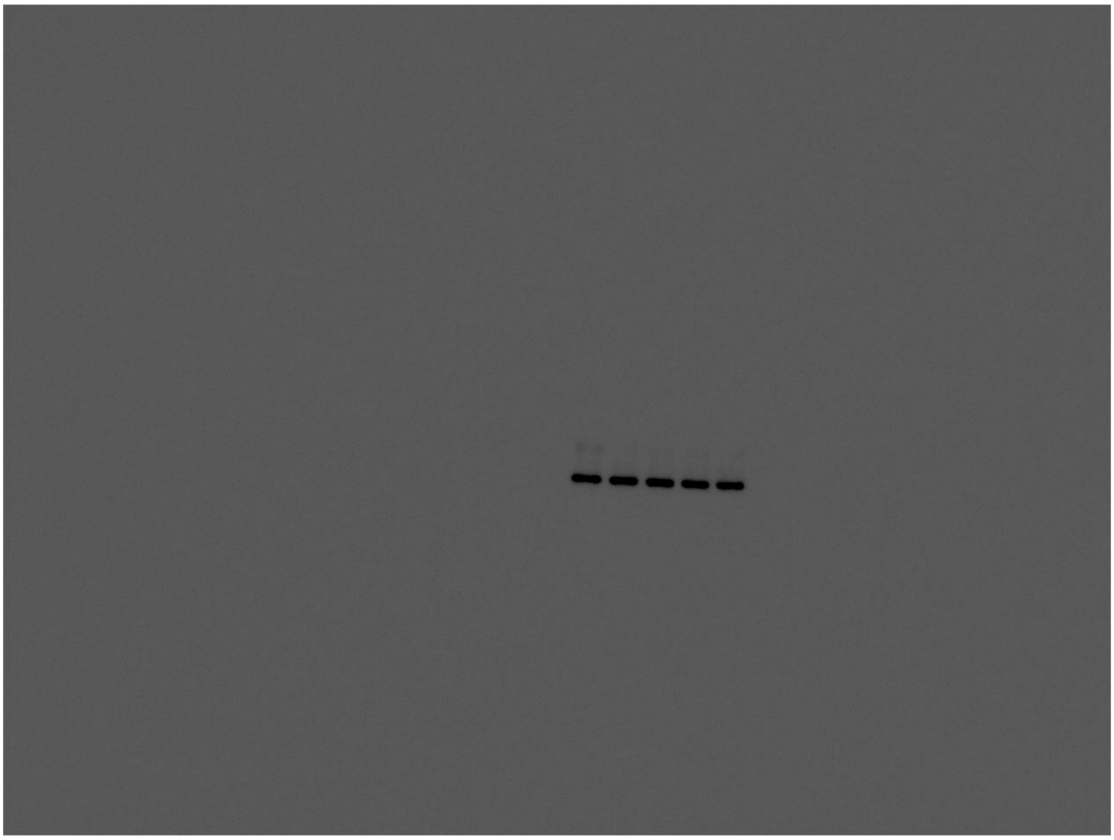

Fig 2
Pp65 1

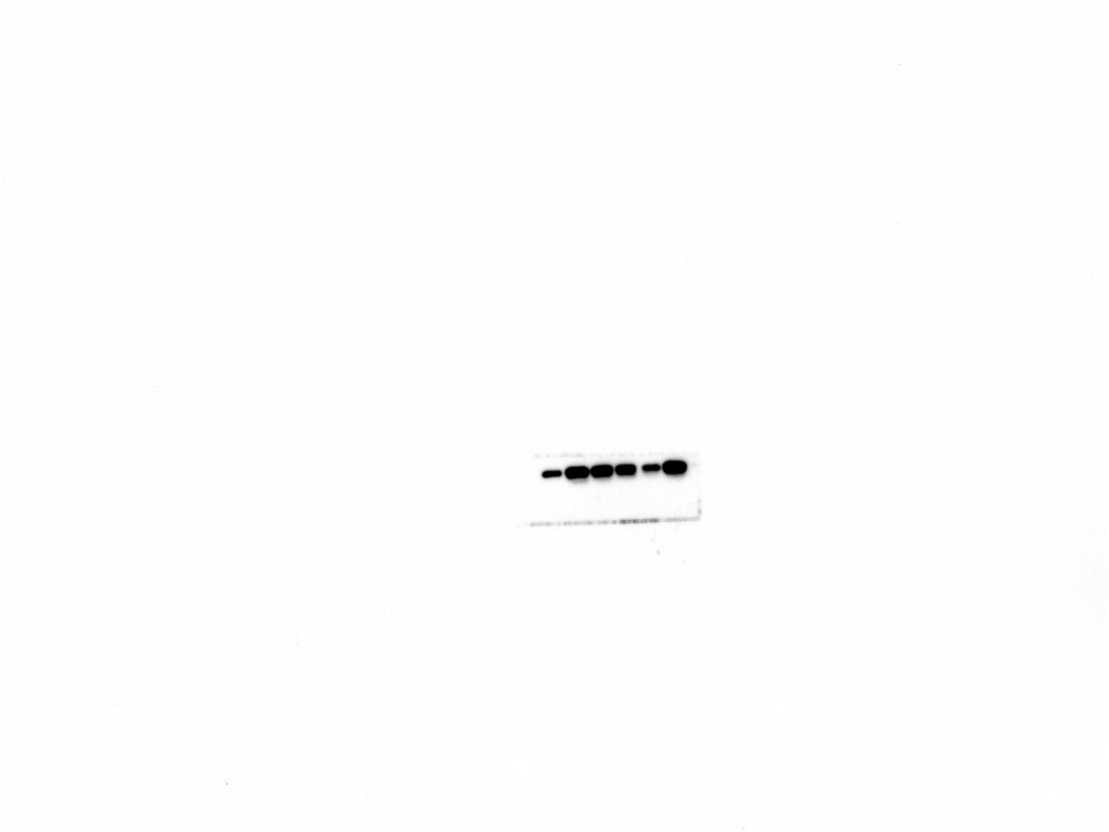

Pp65 2

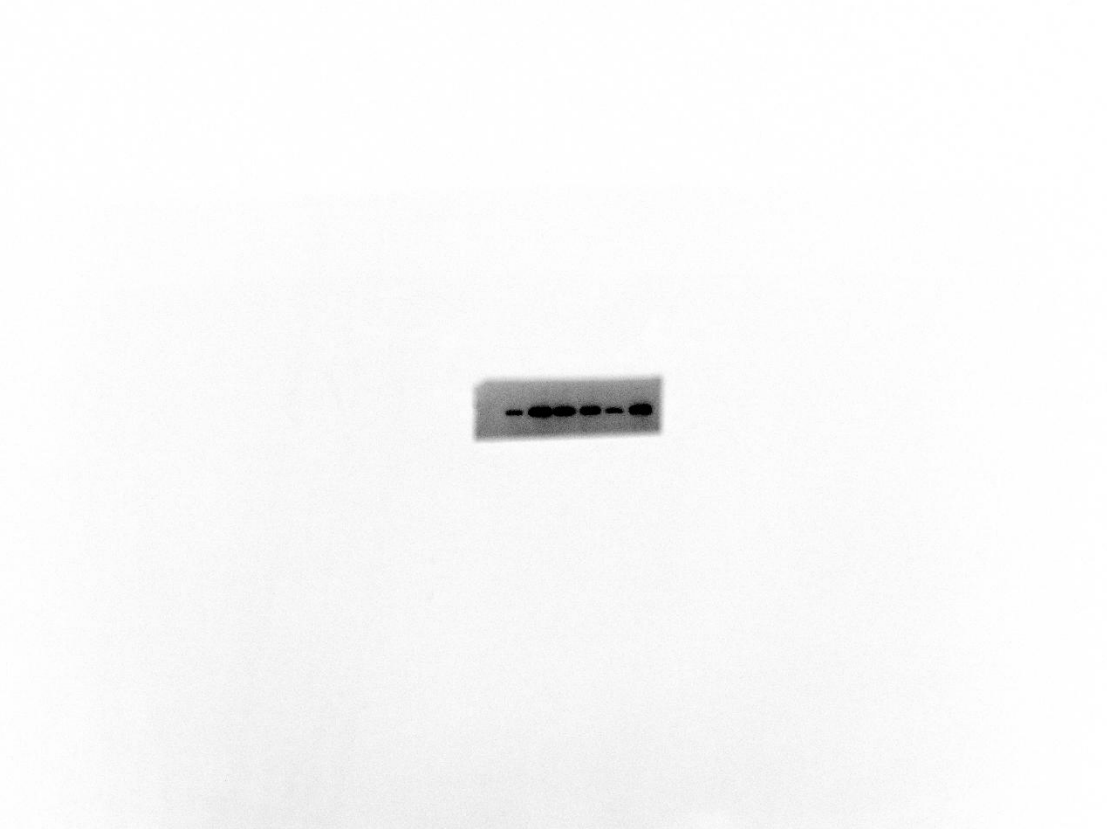

p-p65 3

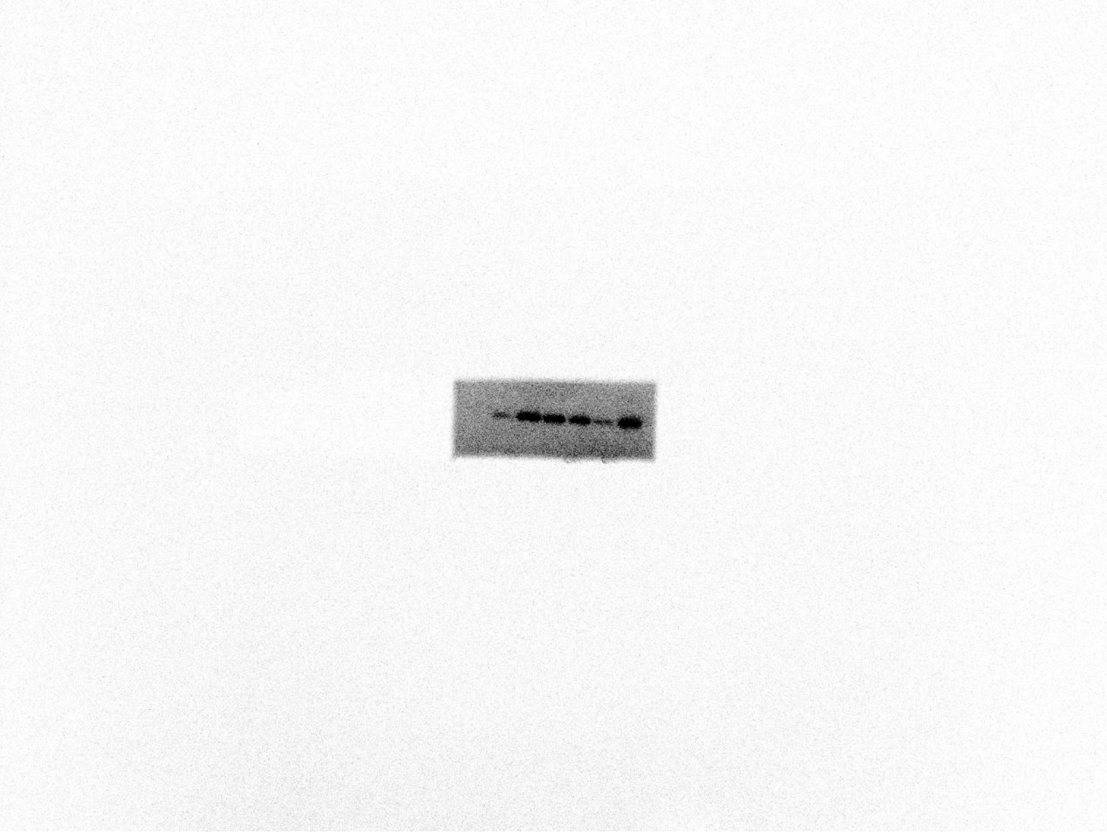

P65 1

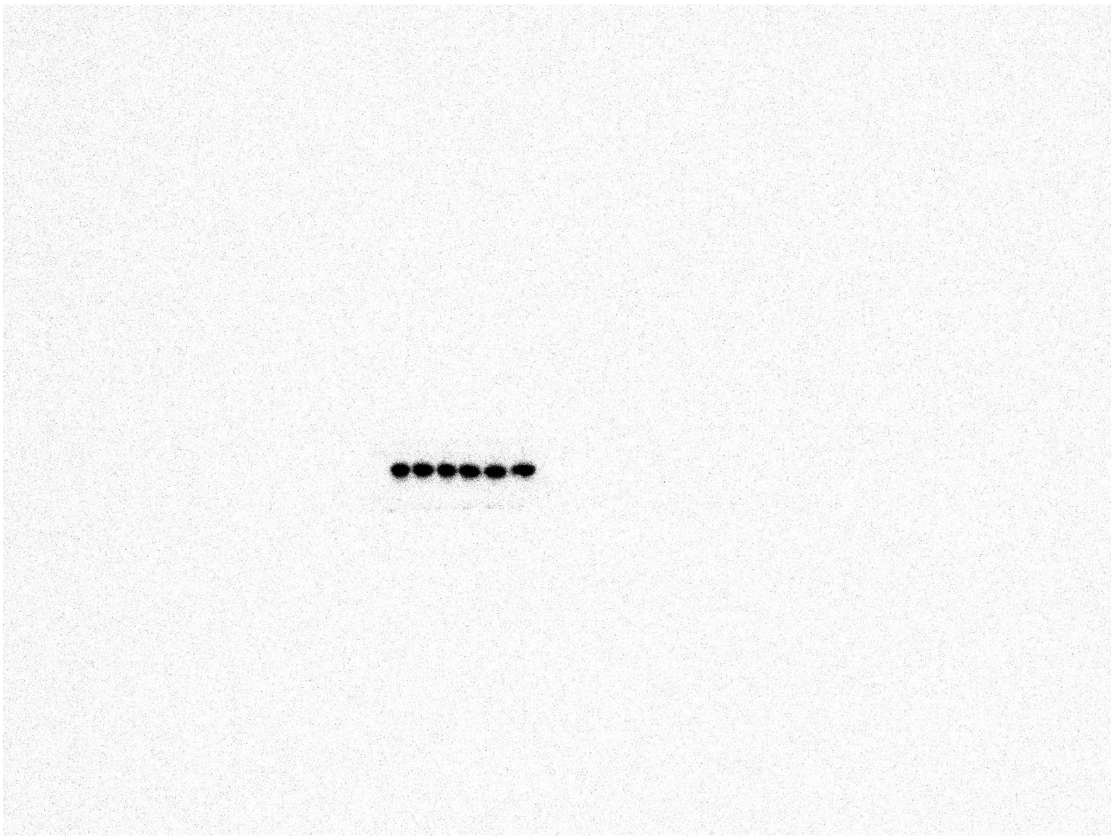

P65 2

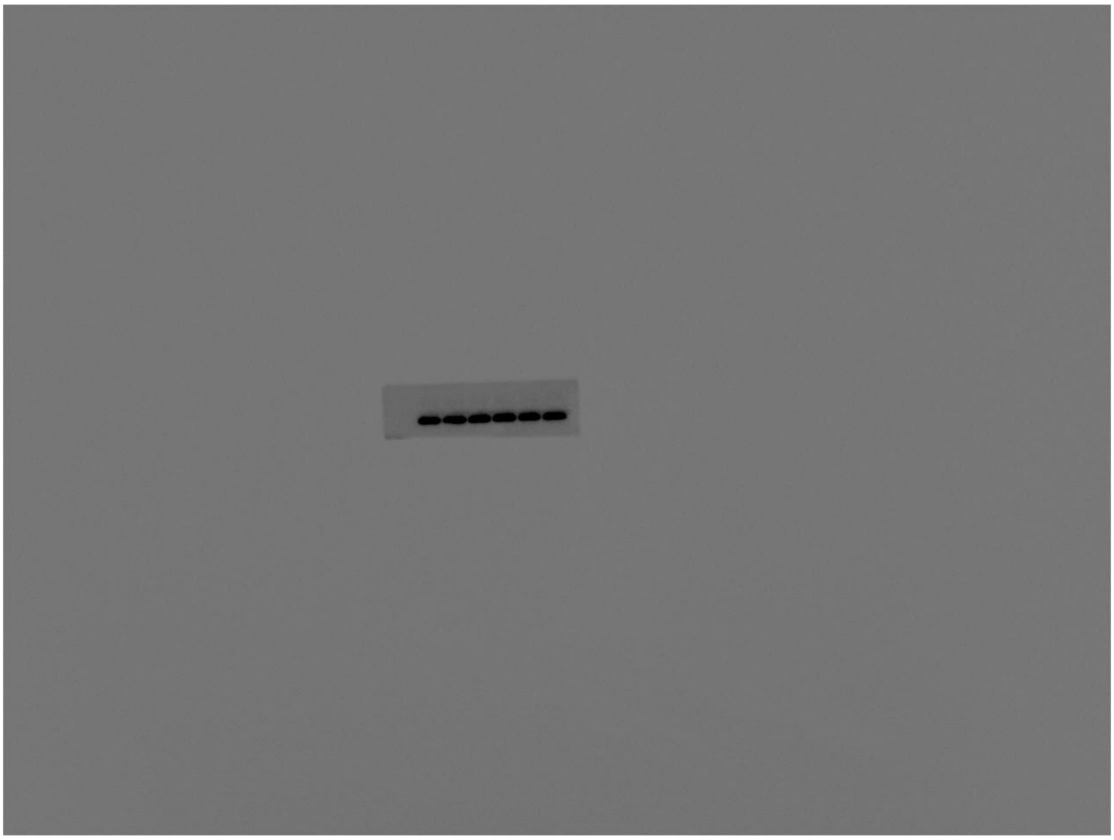

P65 3

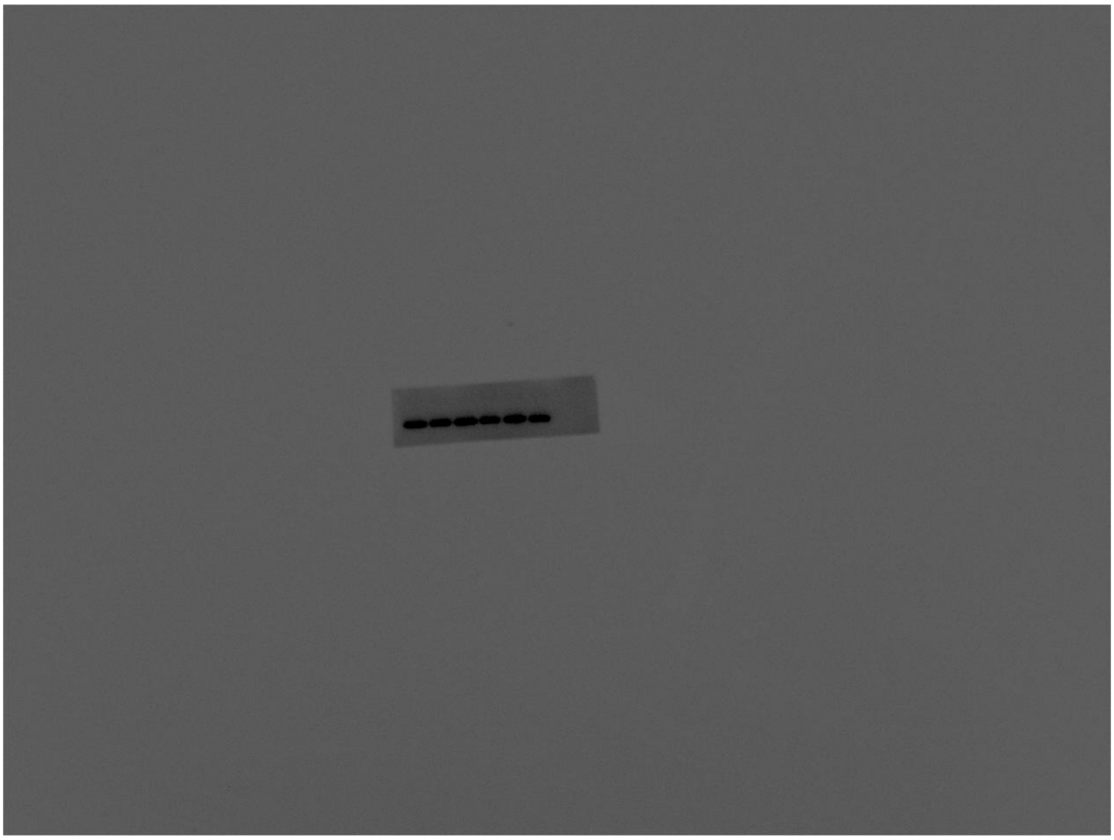

p-IKB 1

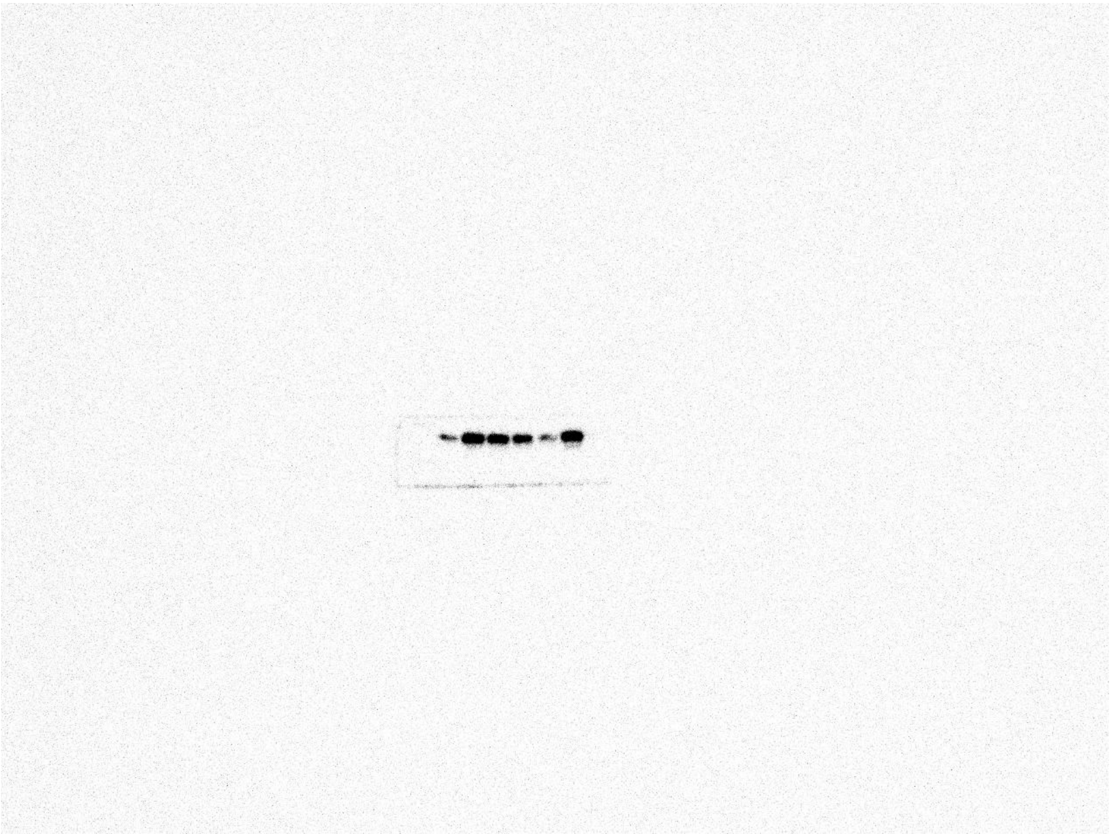

p-IKB 2

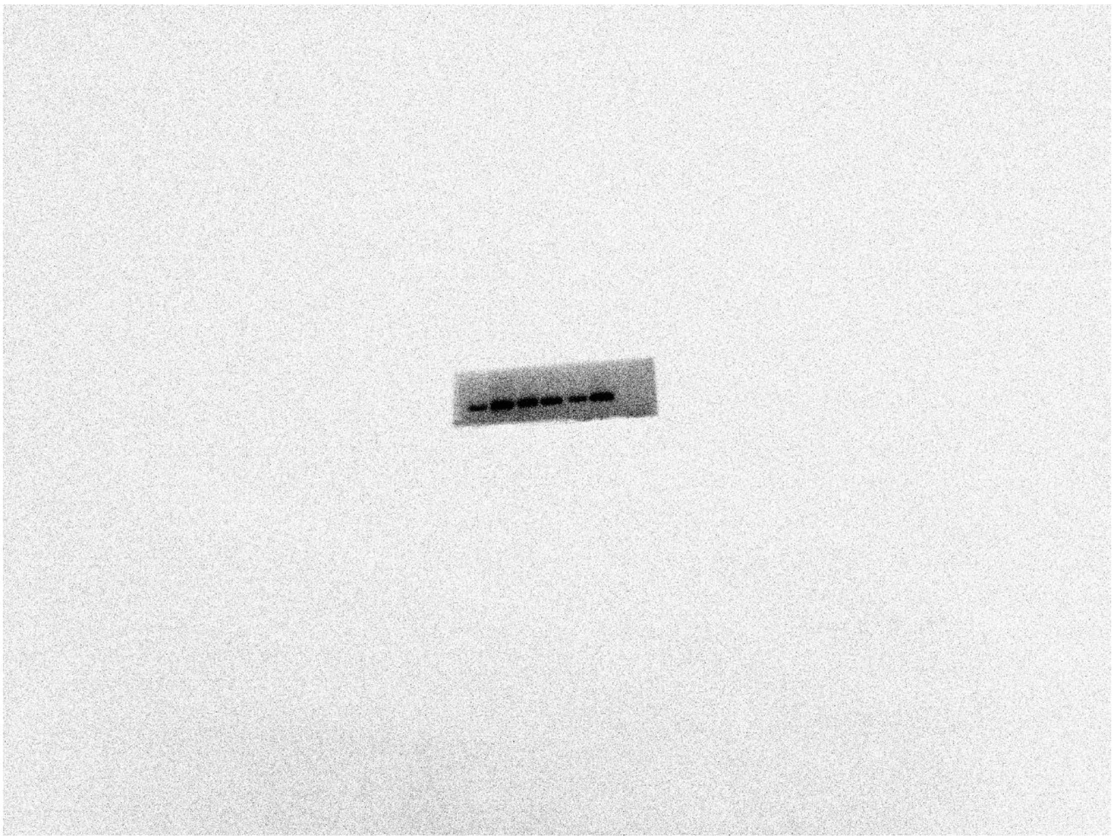

p-IKB 3

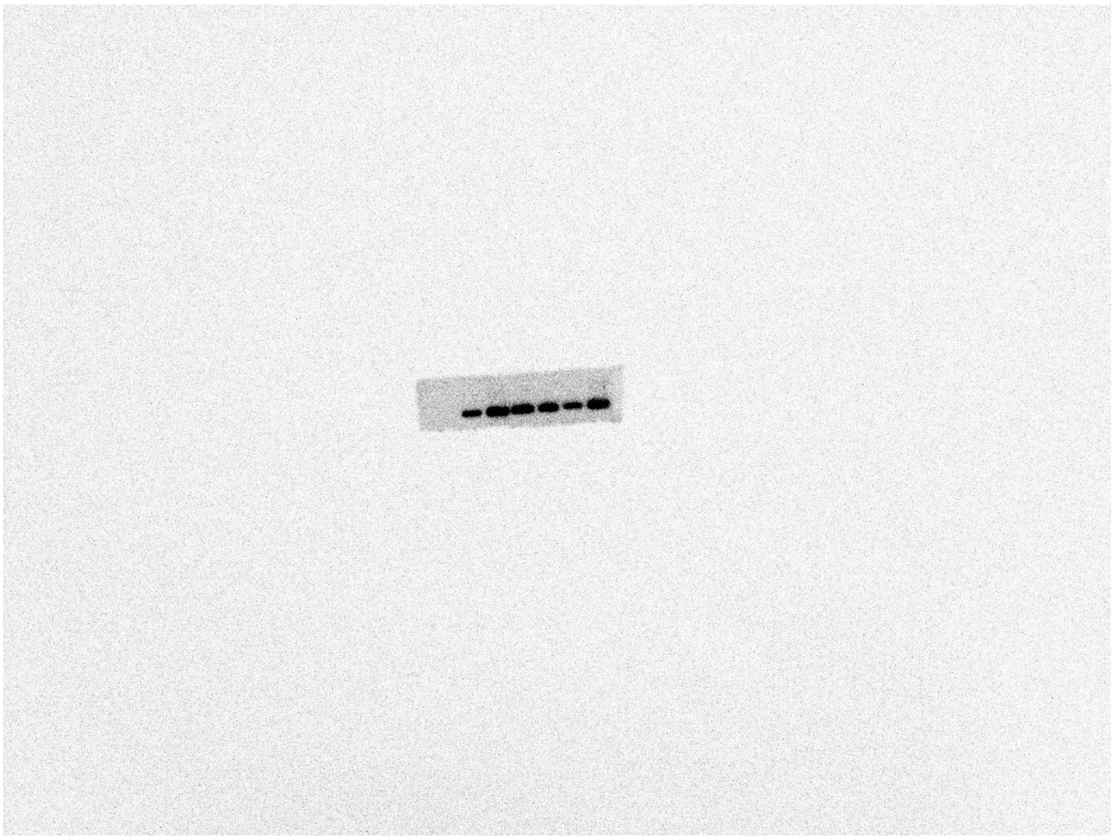

IKB 1

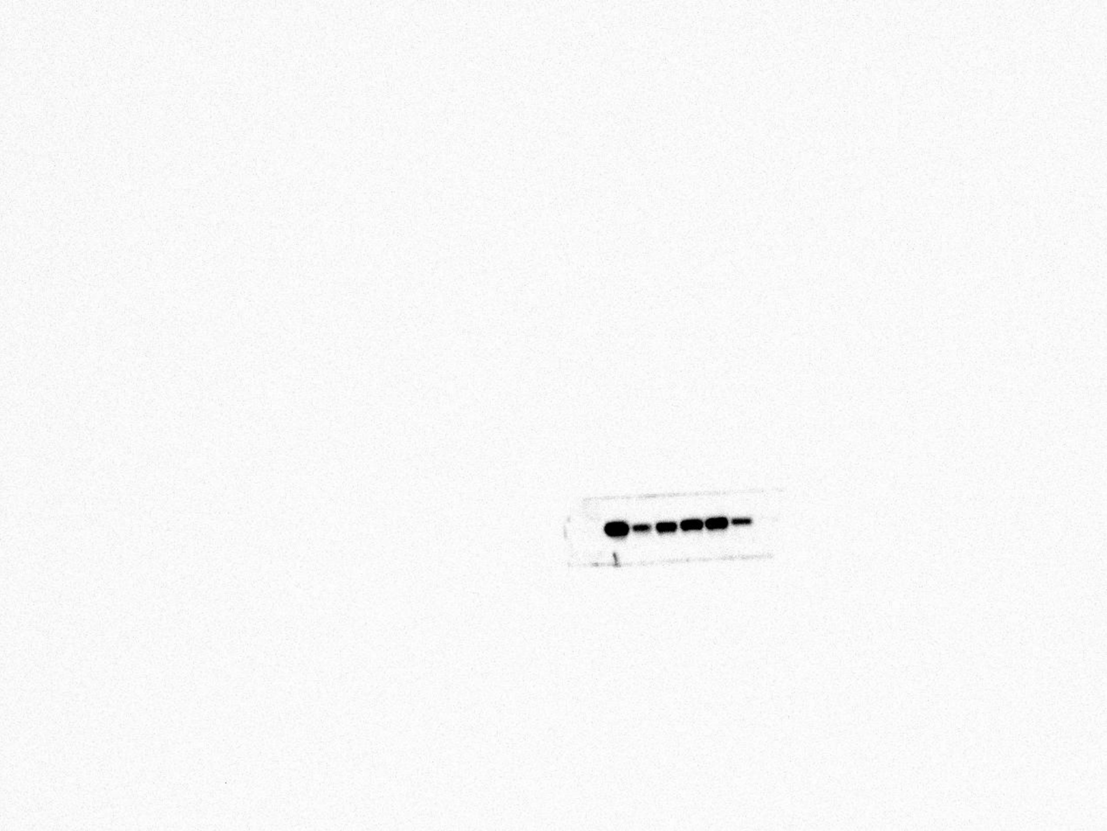

IKb 2

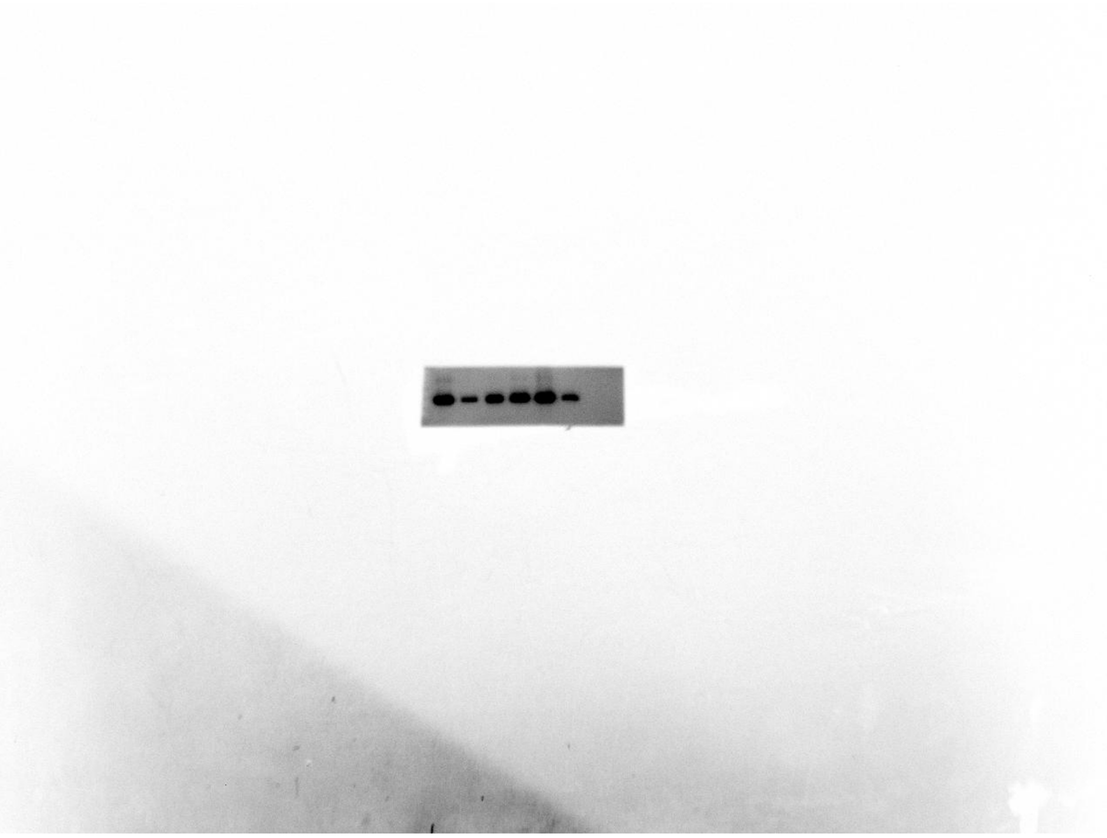

IKB 3

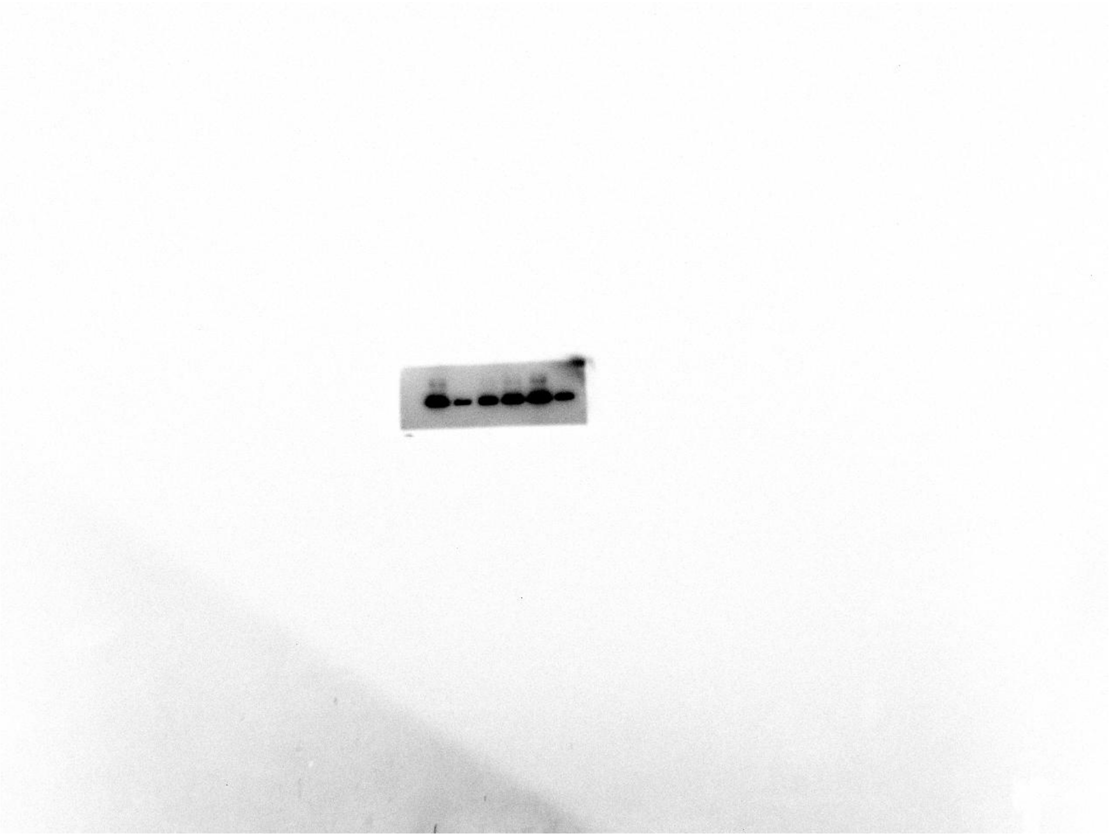

Actin 1

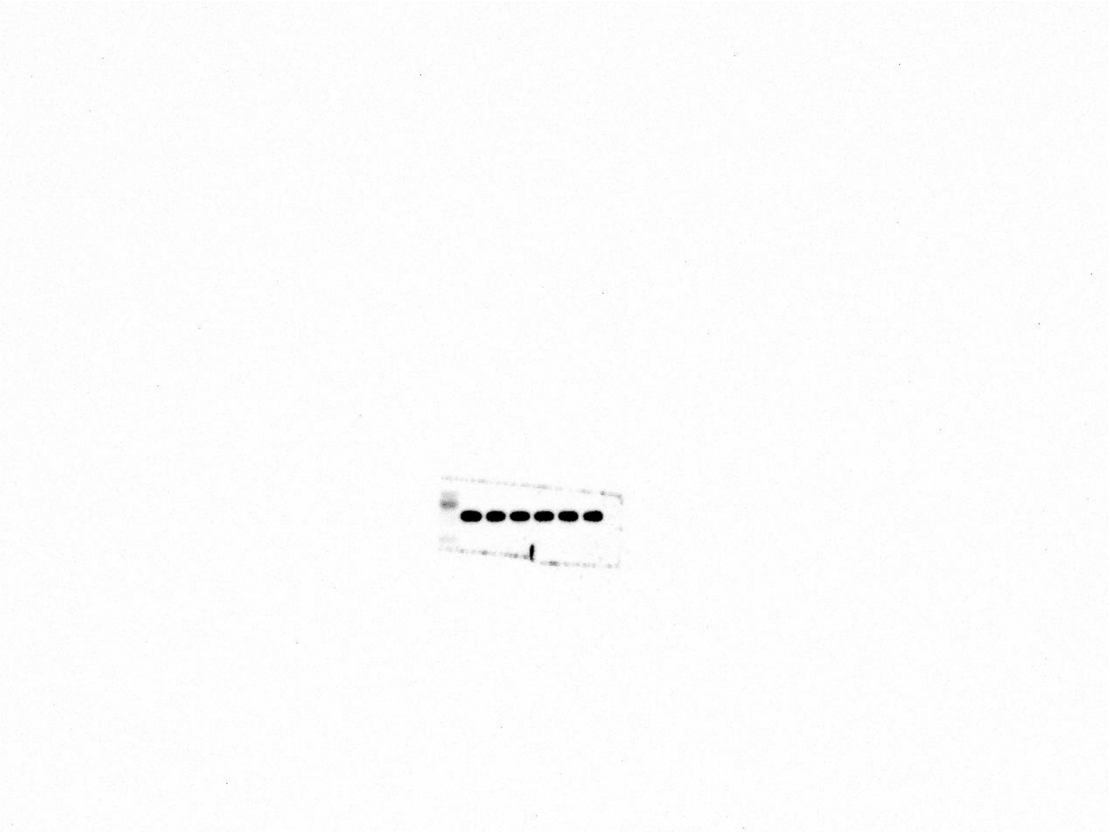

Actin 2

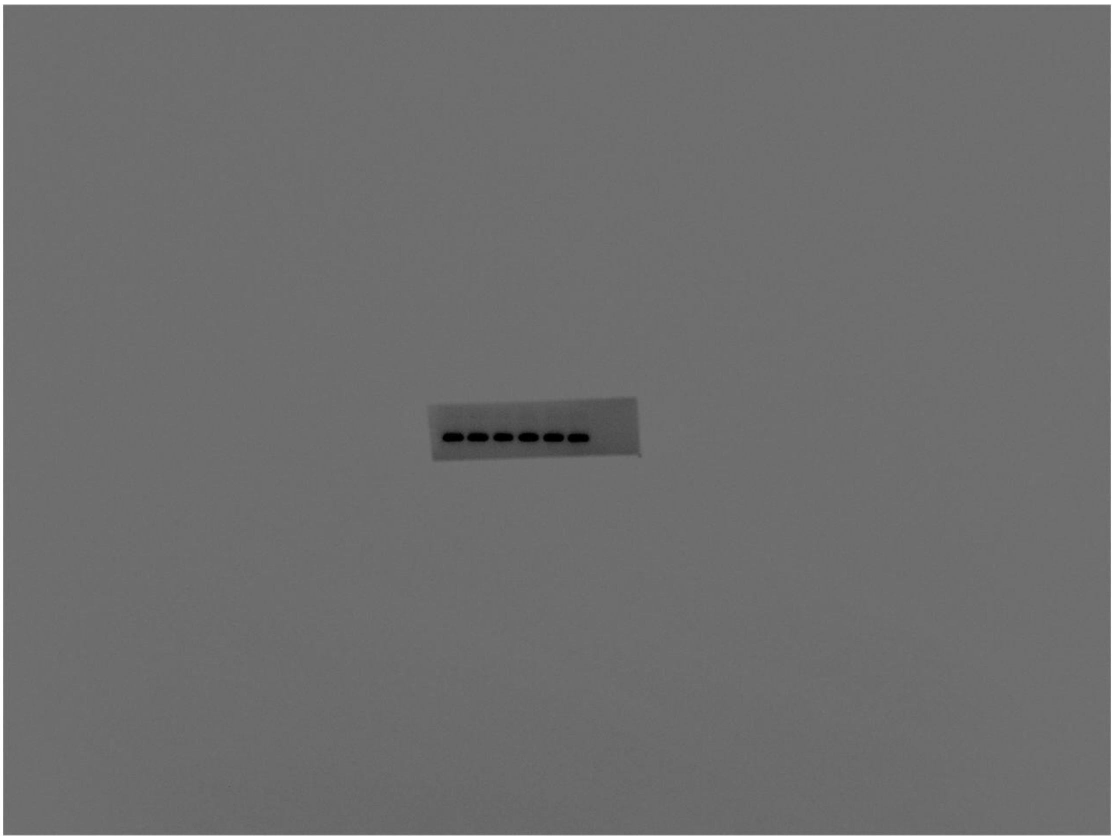

Actin 3

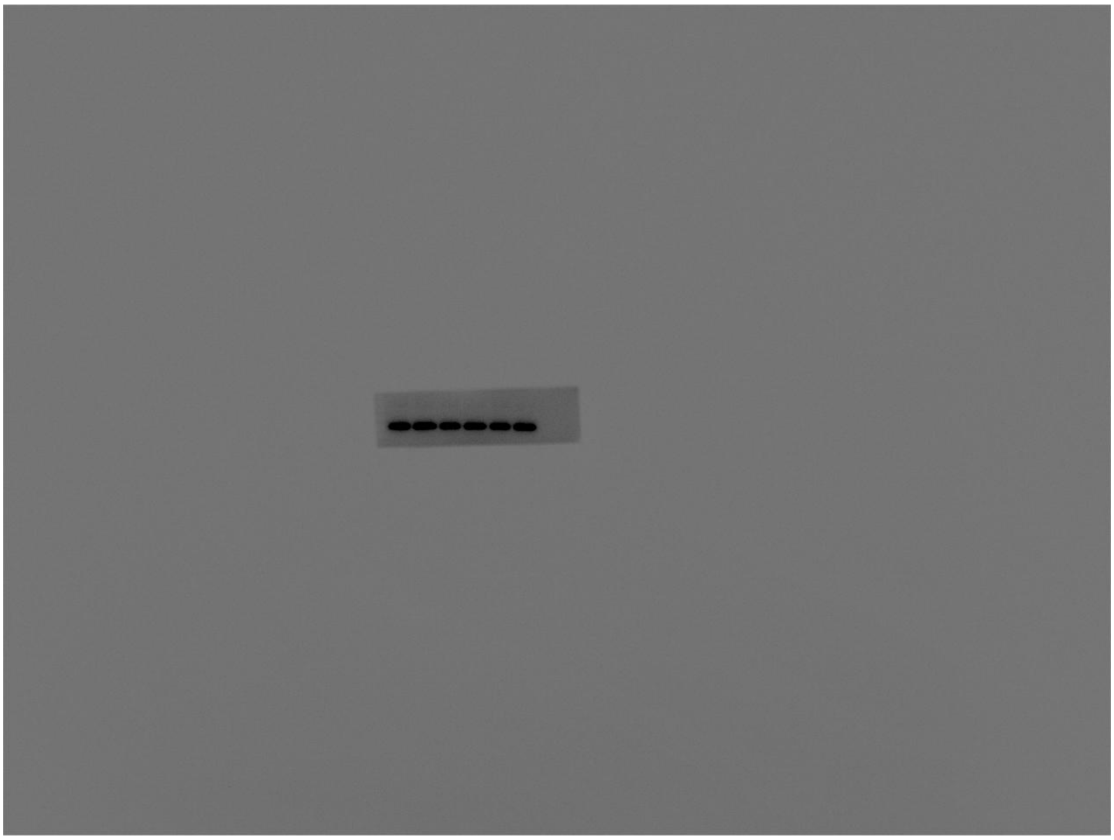

Fig 3

NLRP3 1

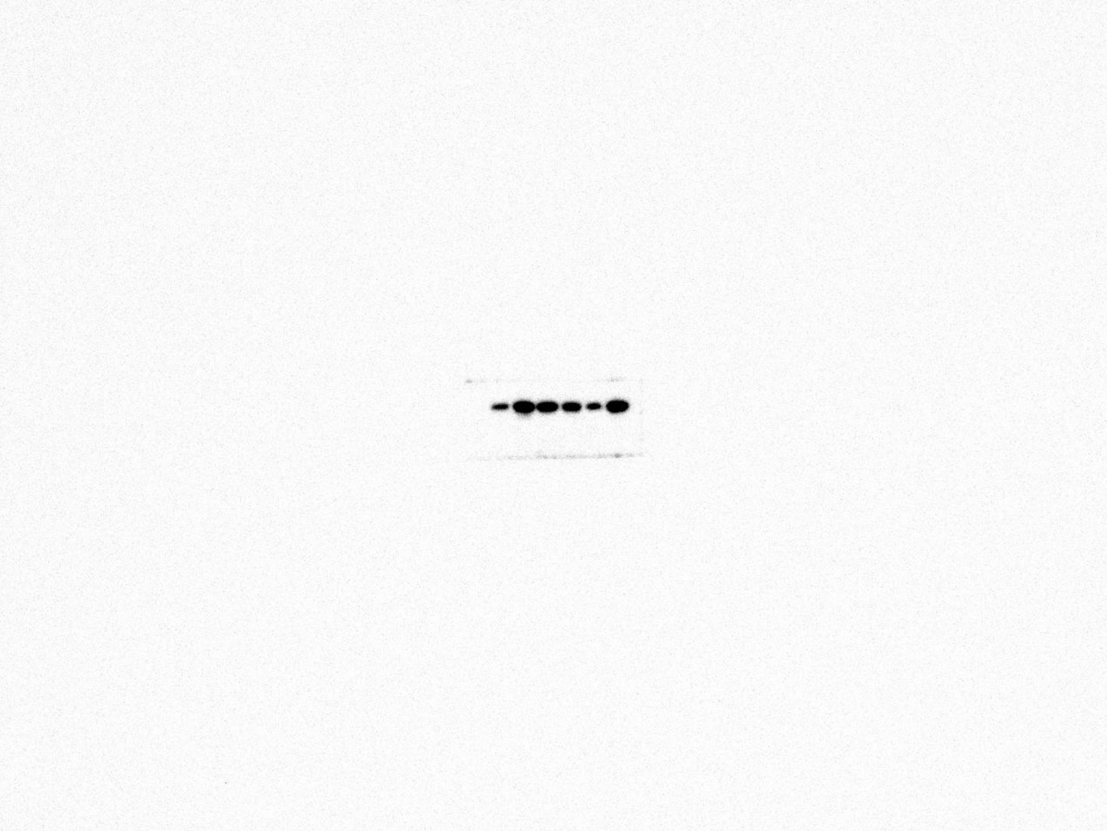

NLRP3 2

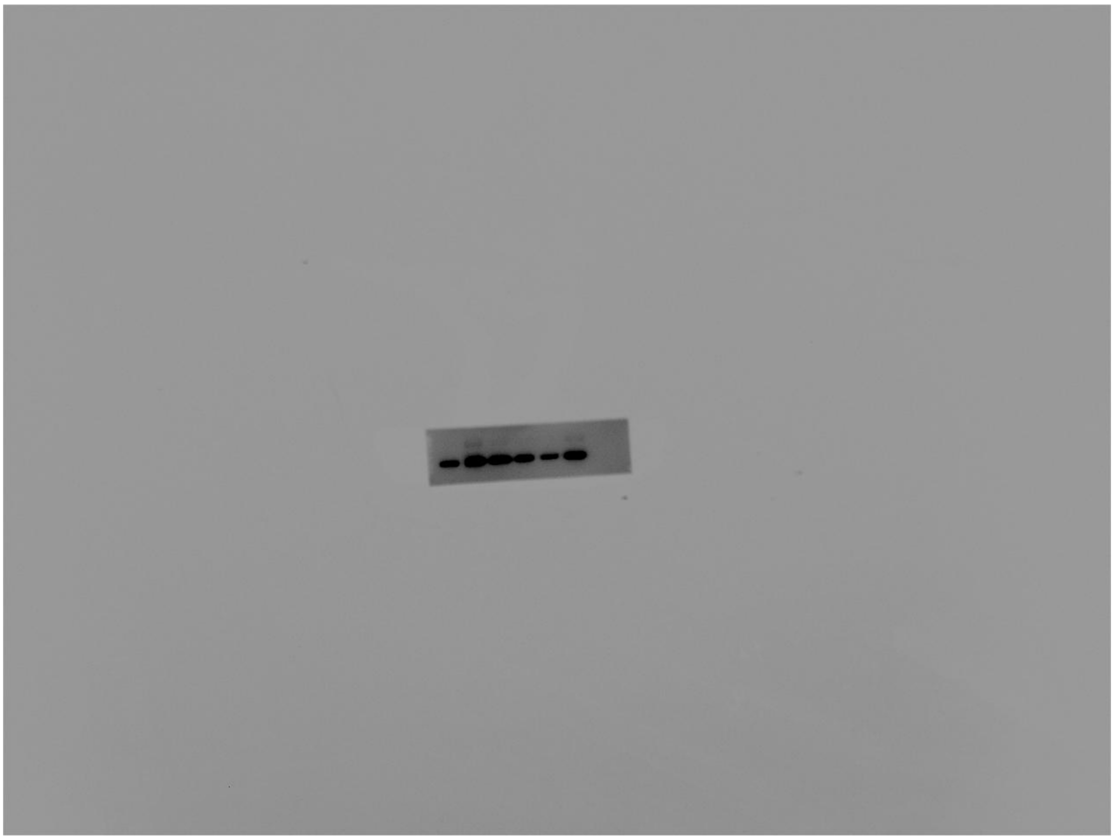

NLRP3 3

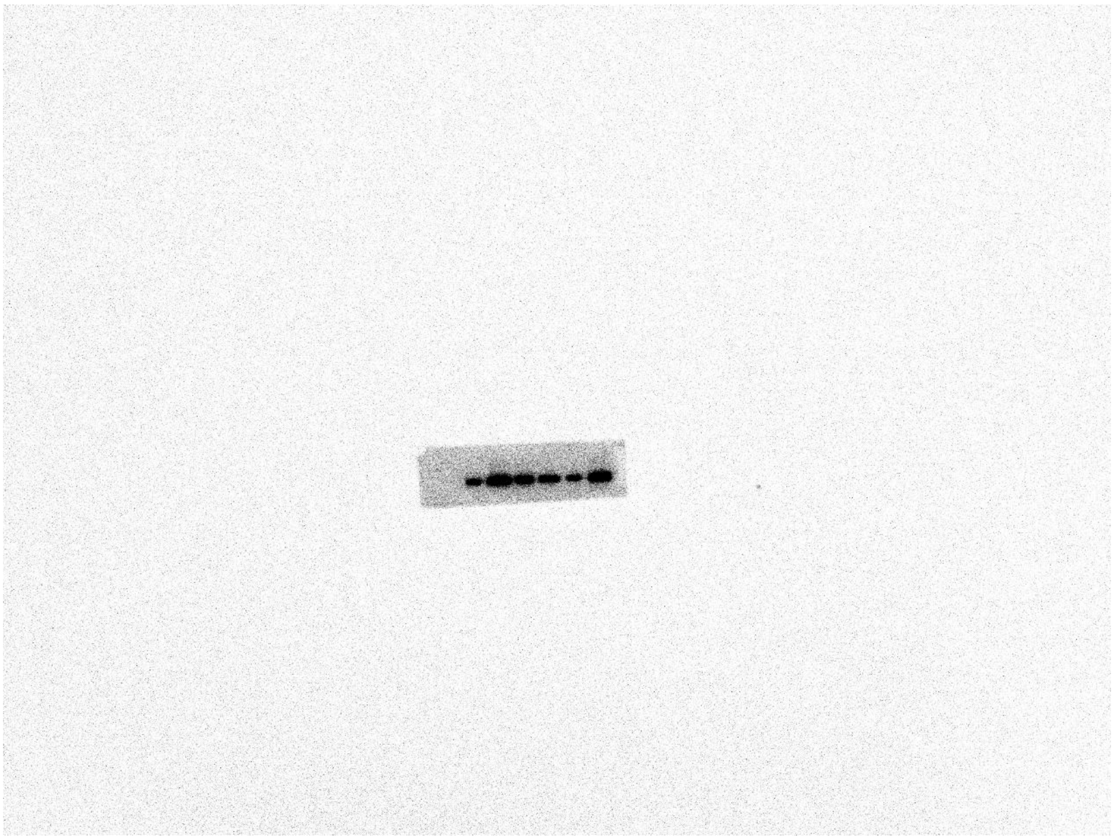

ASC 1

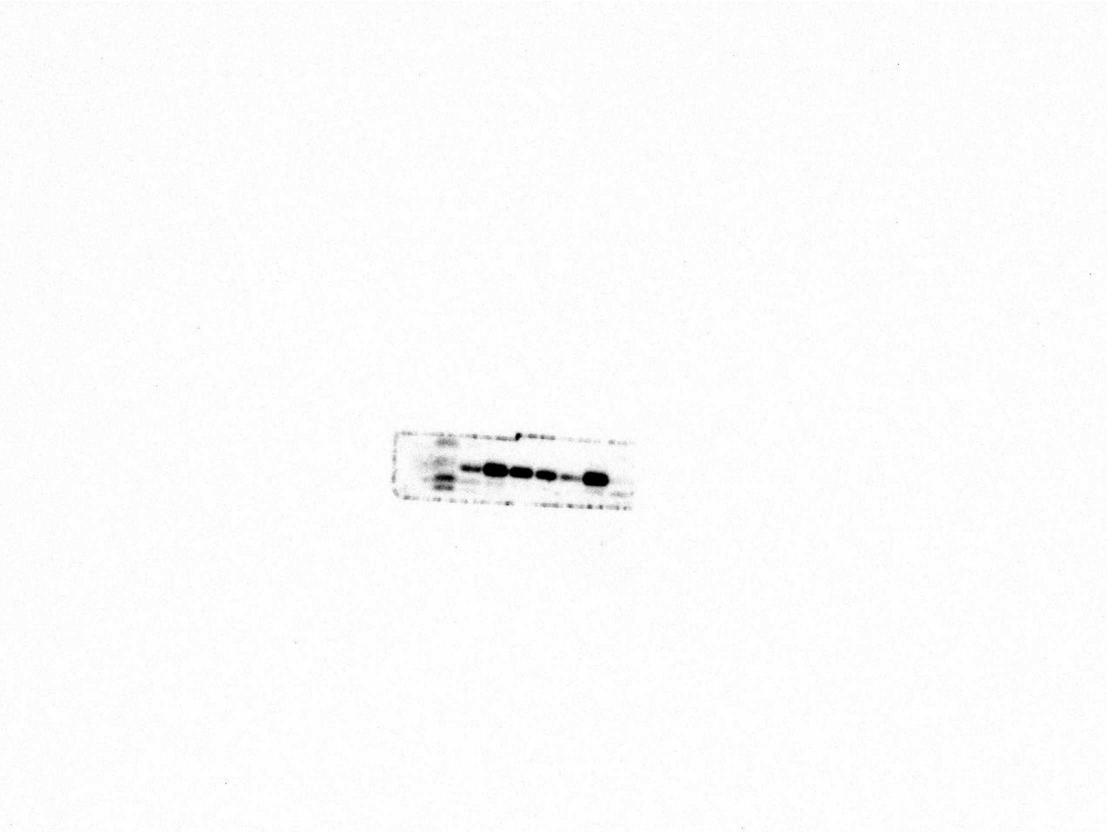

ASC 2

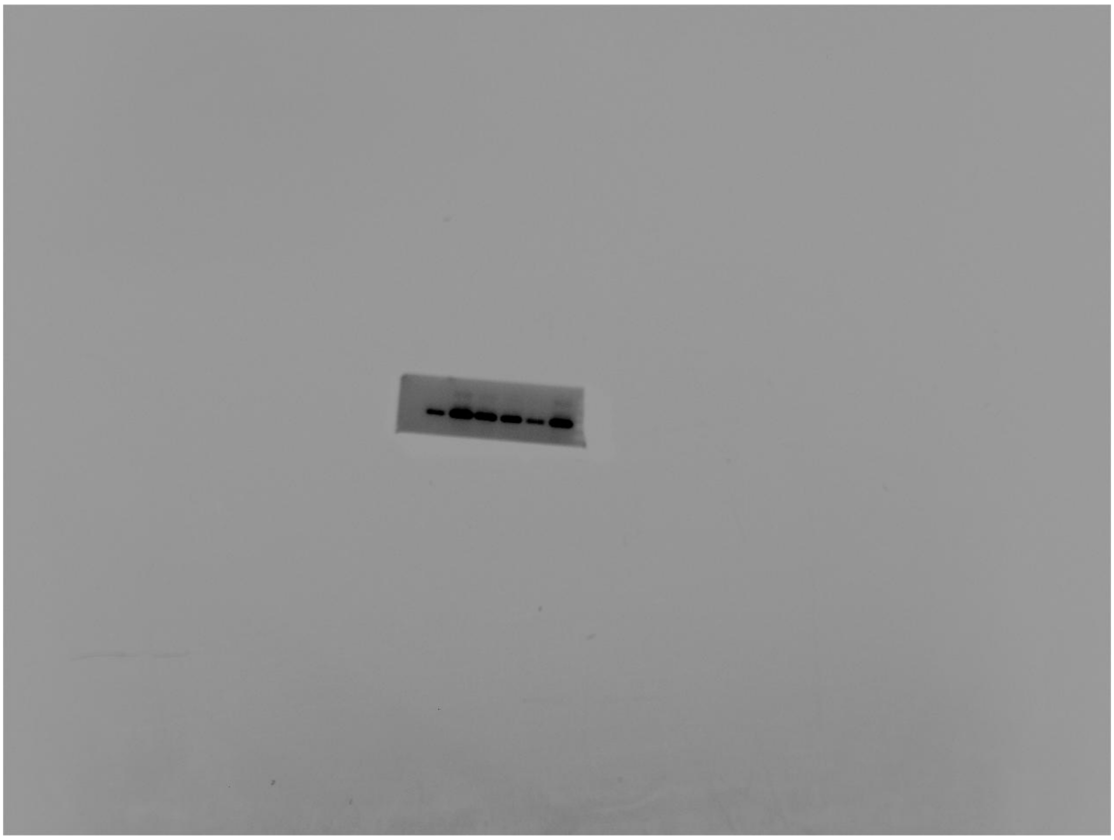

ASC 3

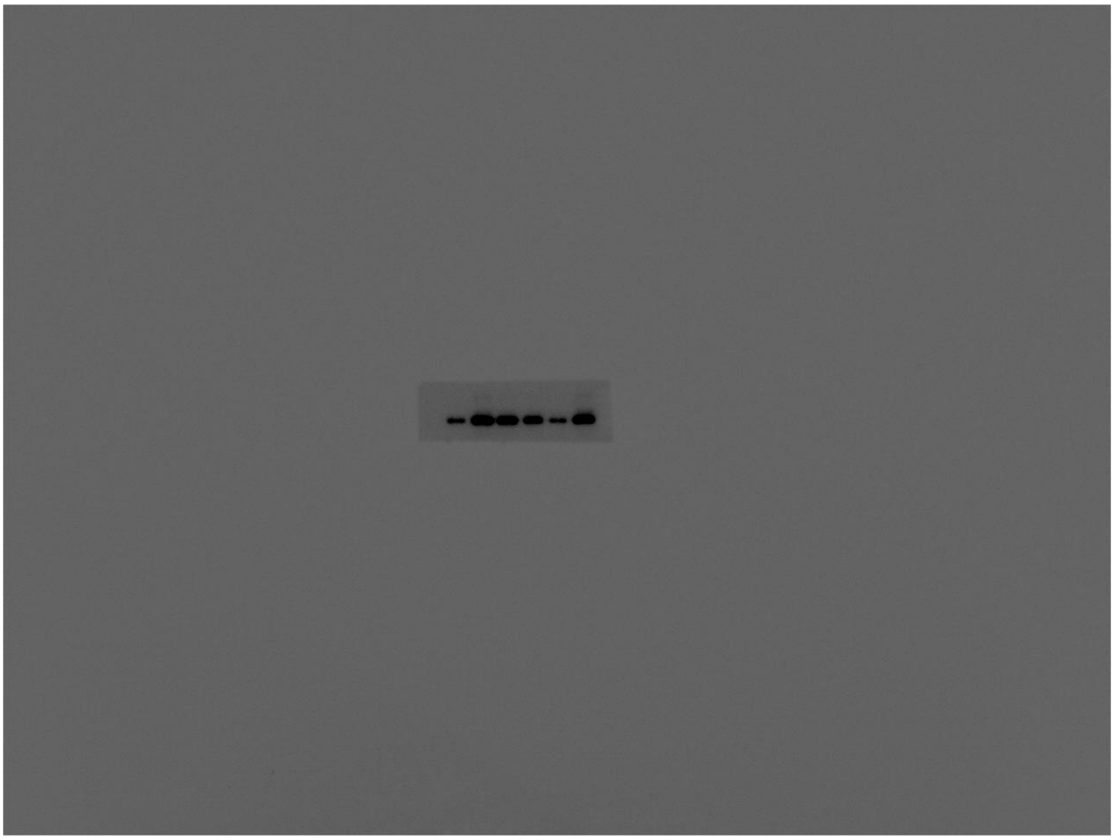

Cas 1 1

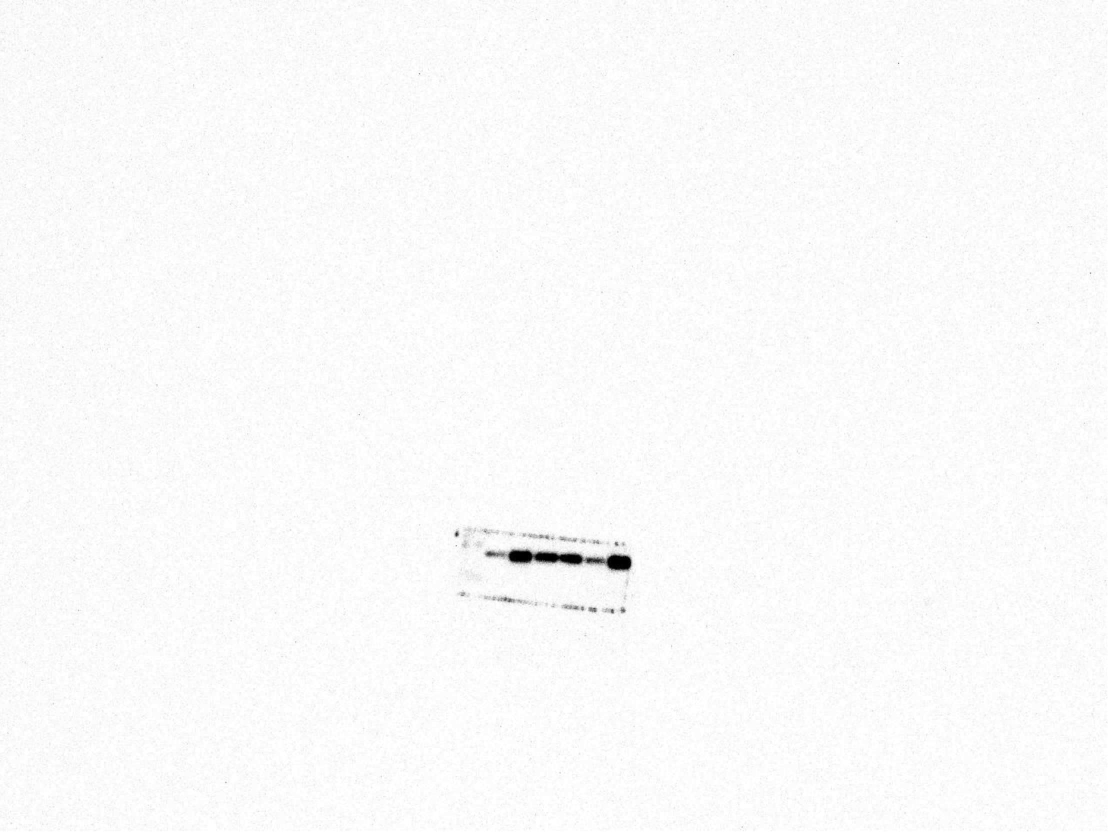

Cas 1 2

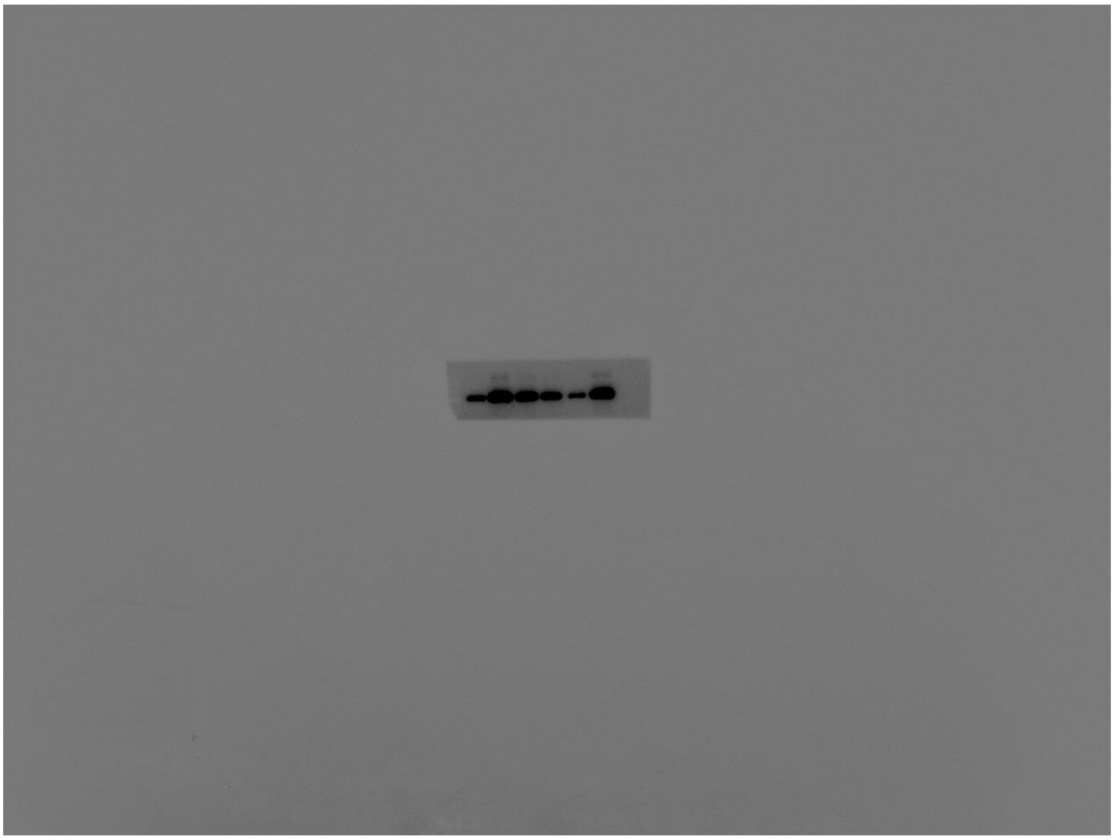

Cas 1 3

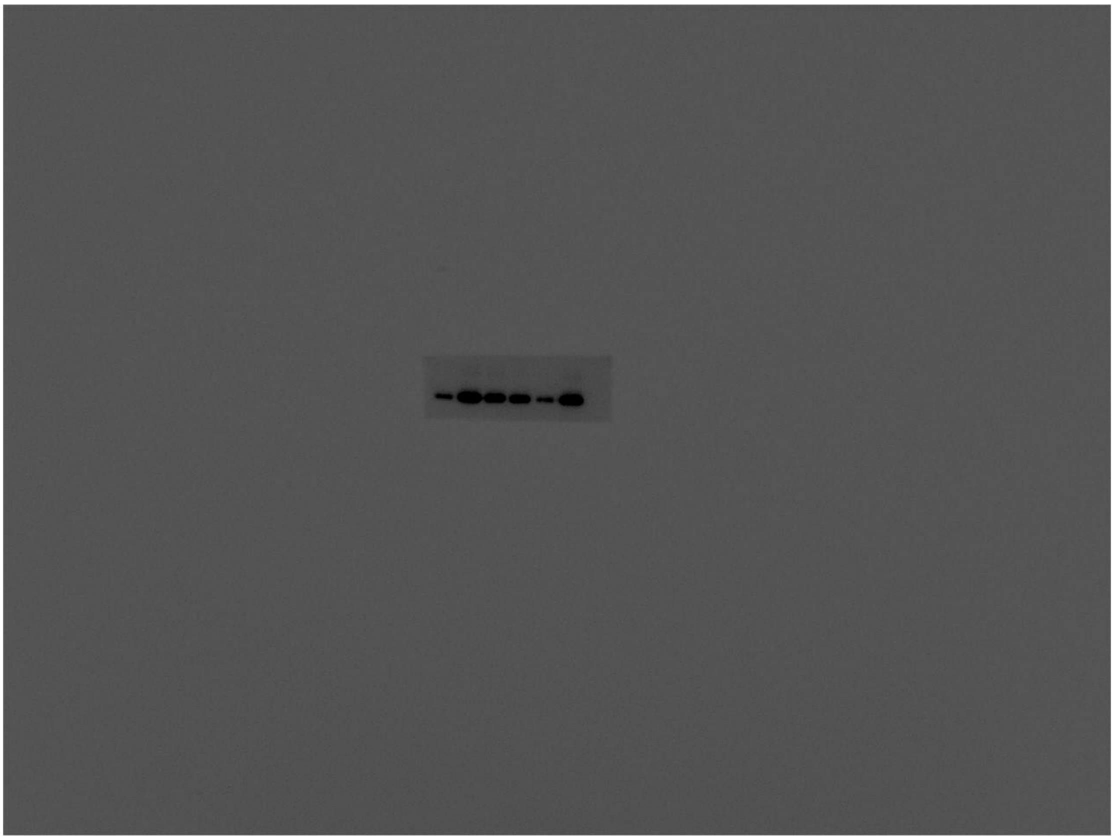

Actin 1

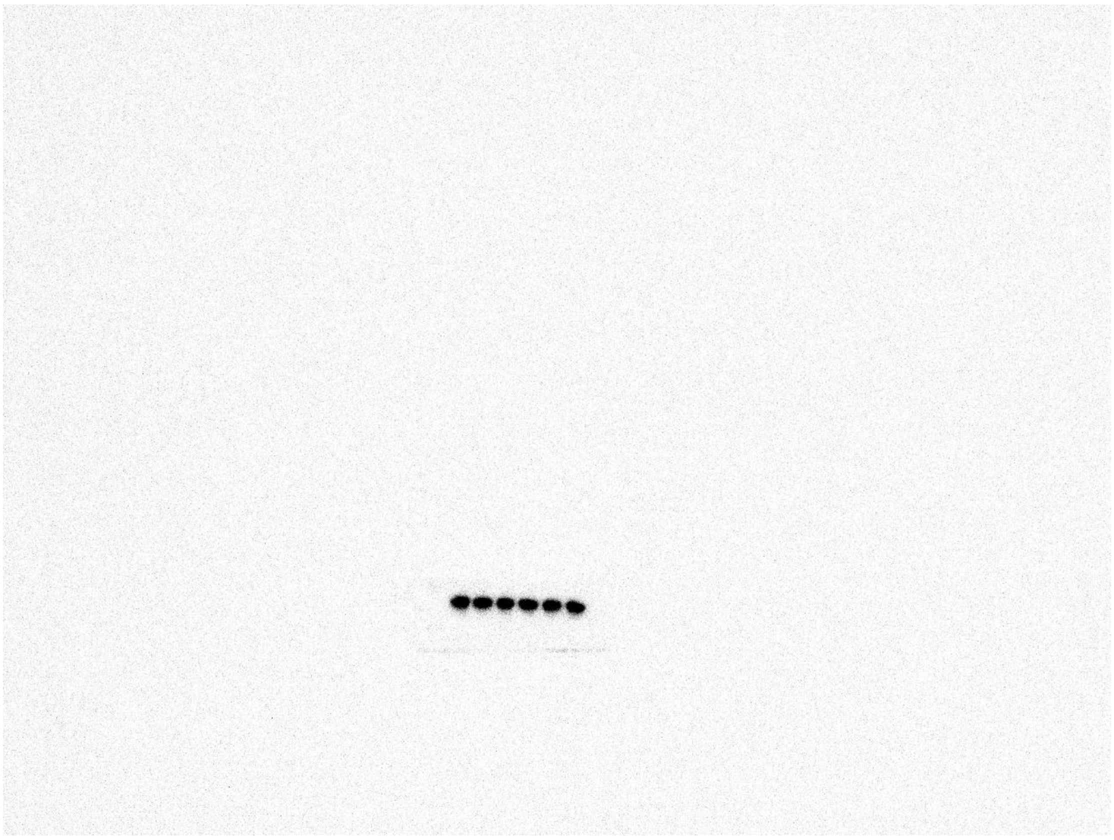

Actin 2

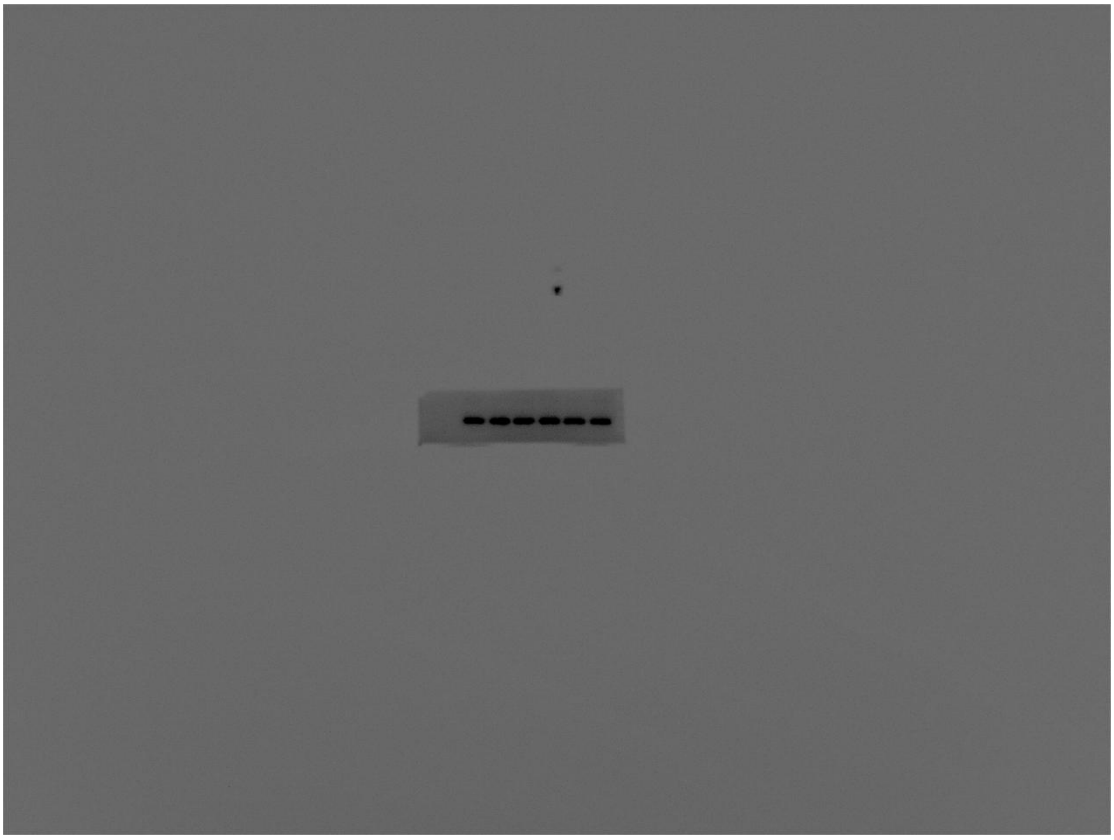

Actin3

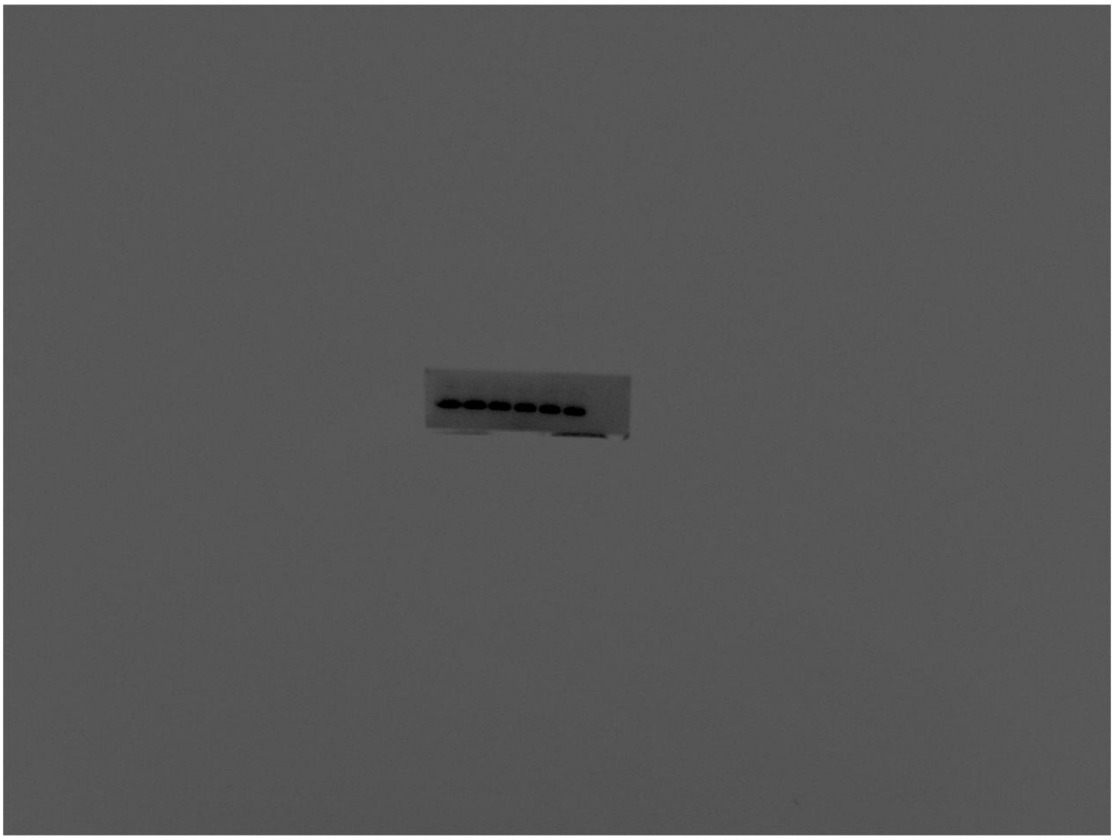

Fig 4

AhR 1

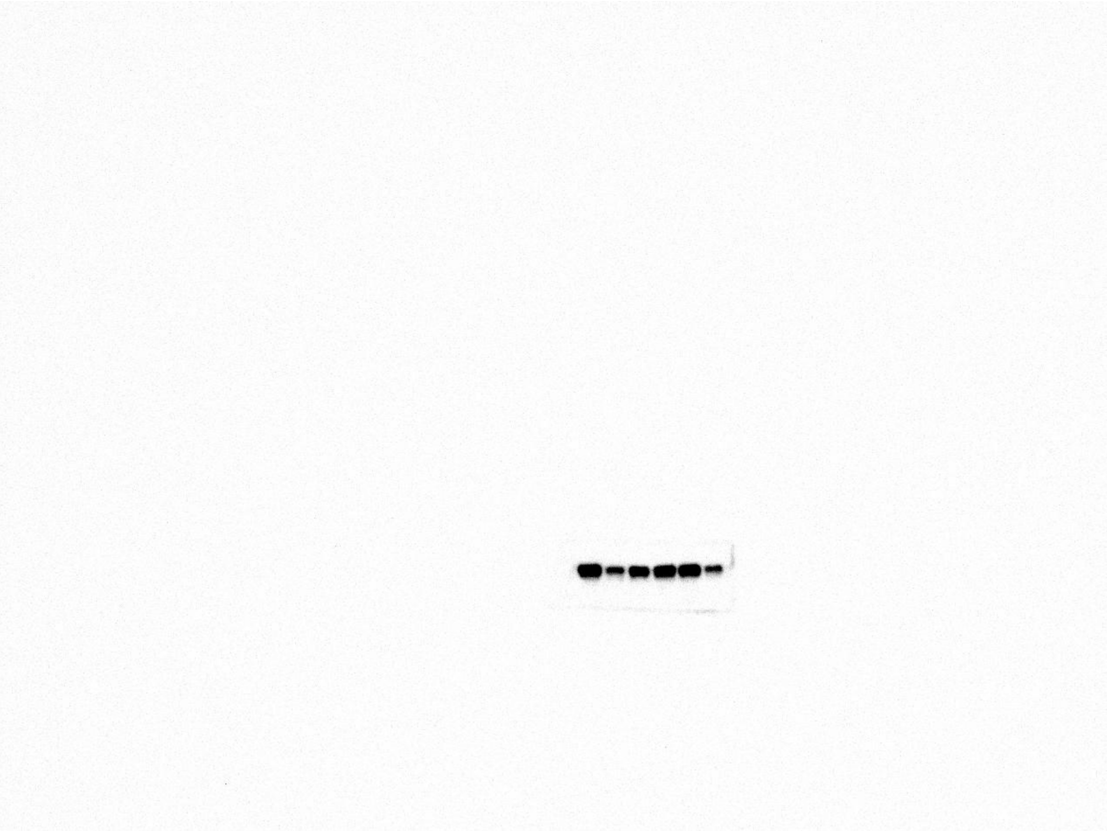

AhR 2

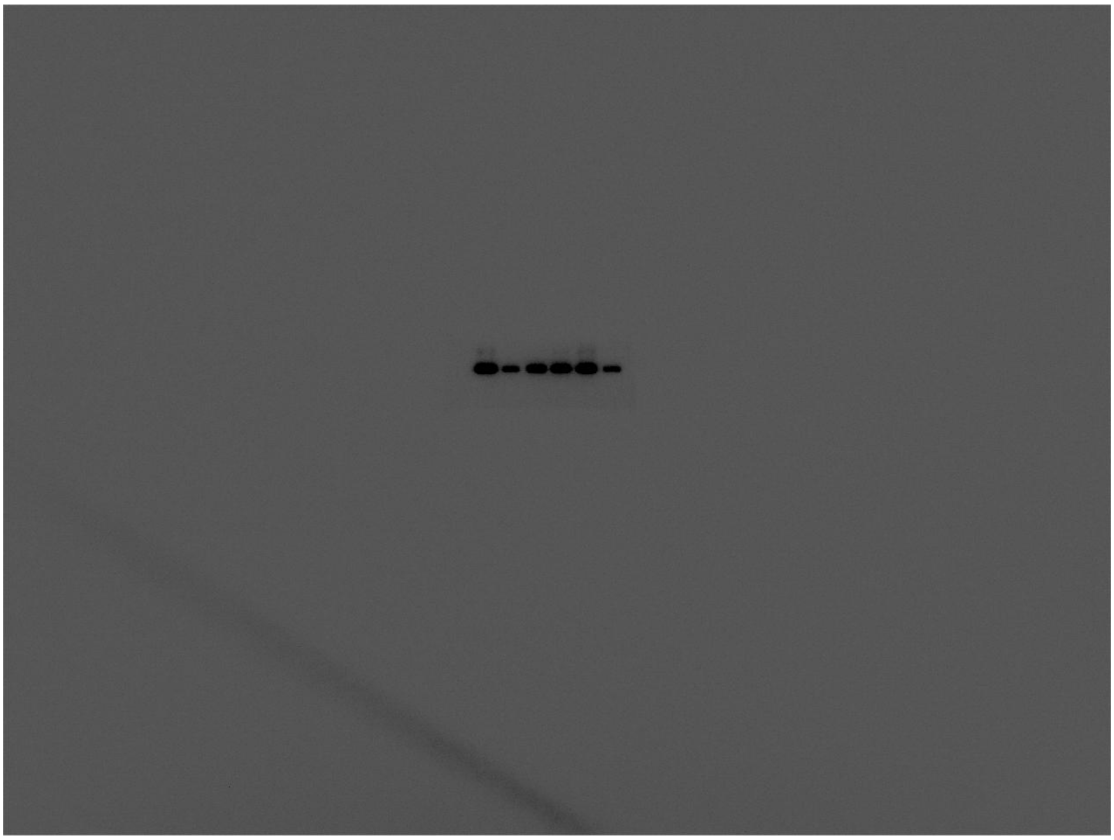

AhR 3

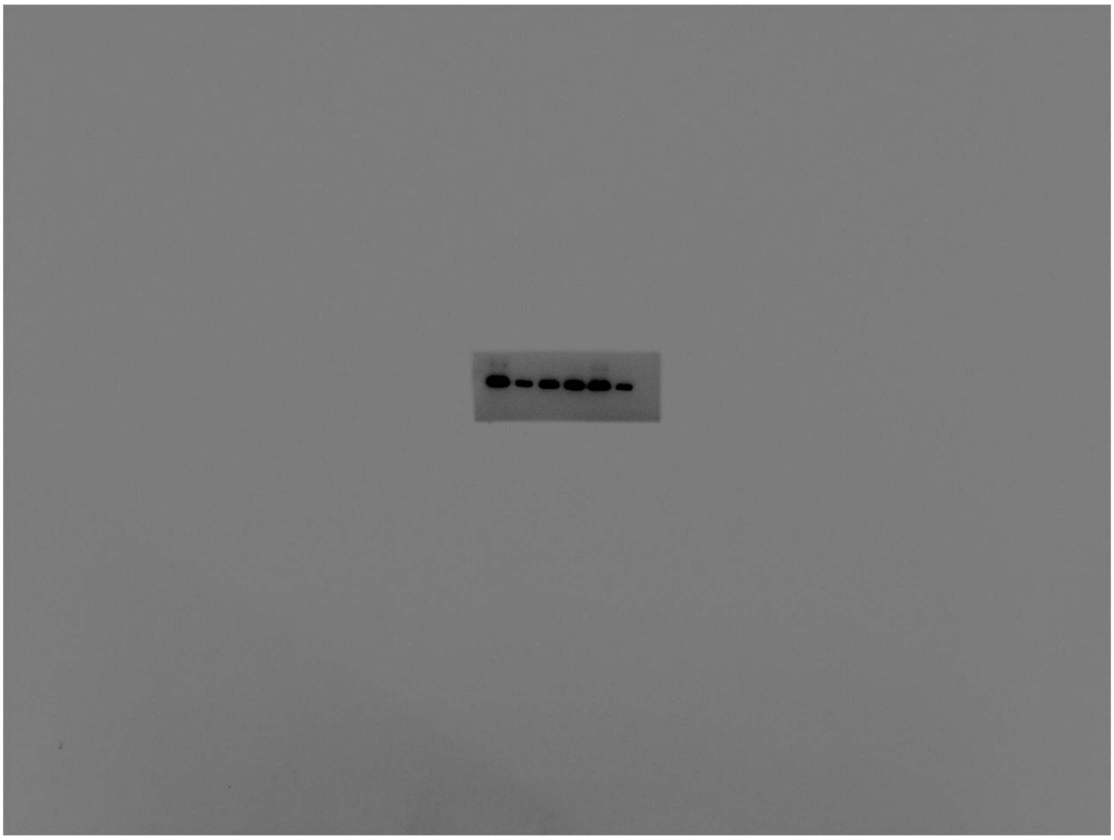

Actin 1

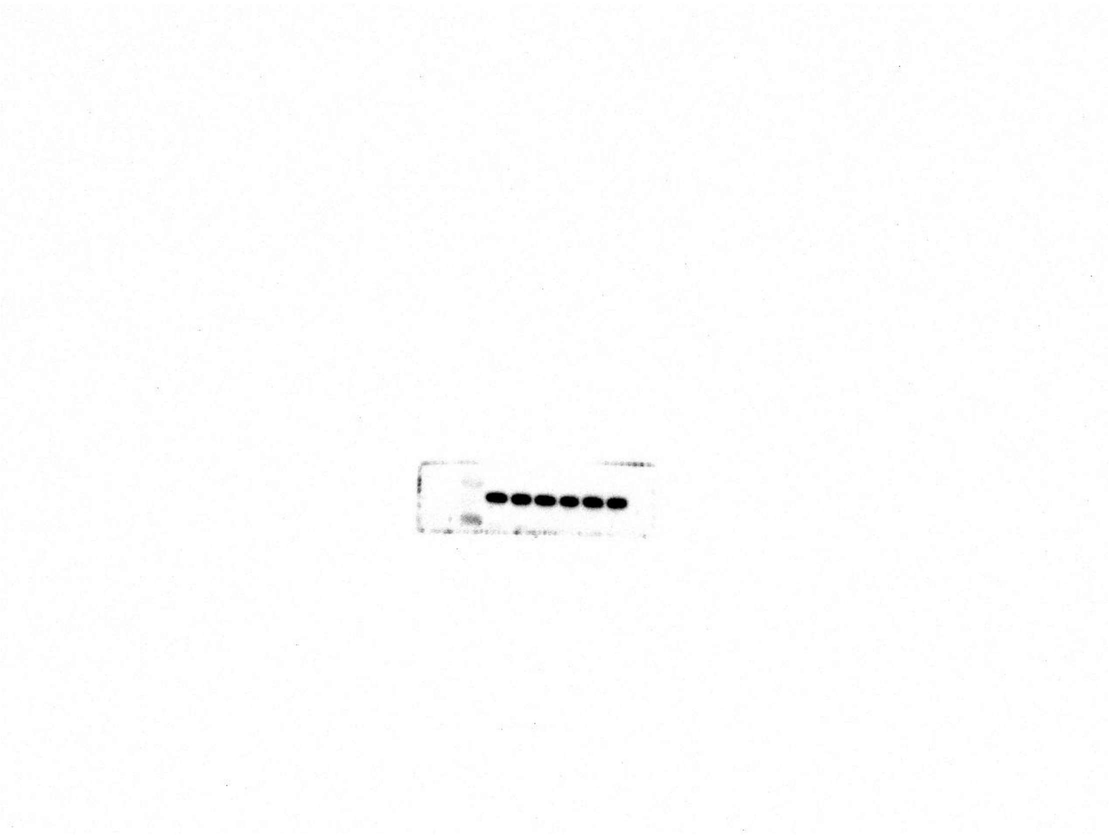

Actin 2

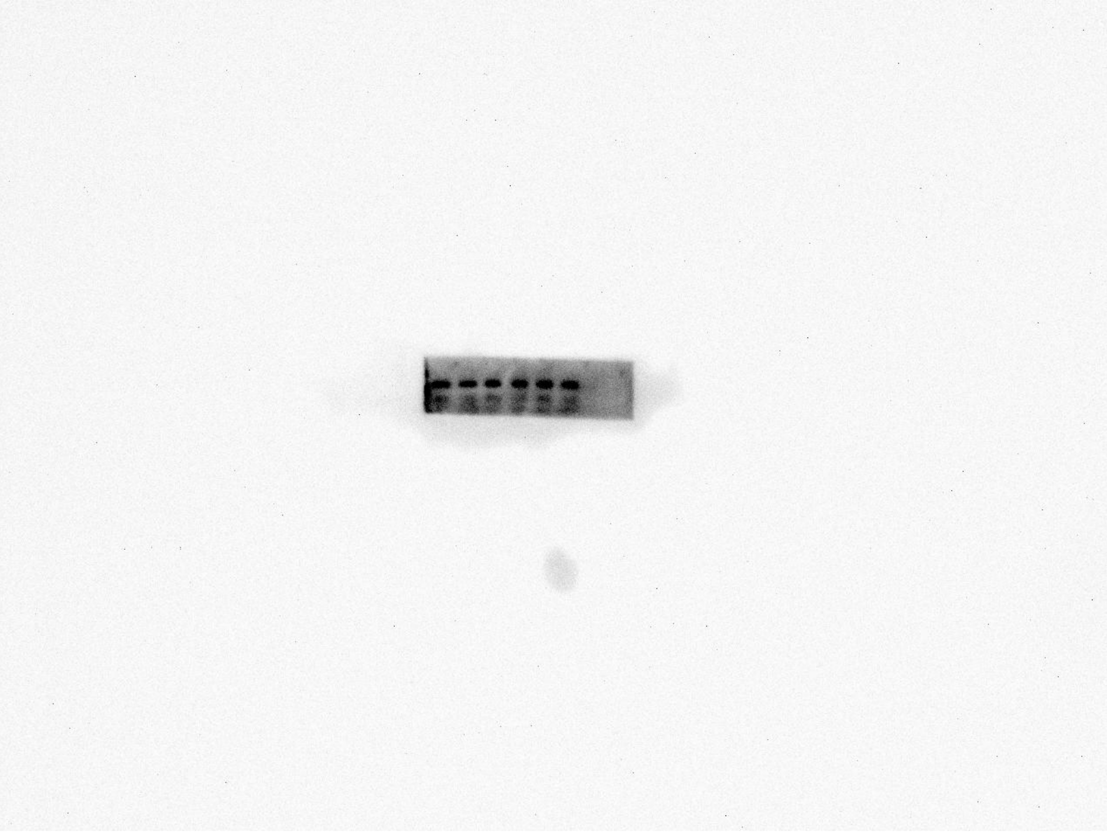

Actin3

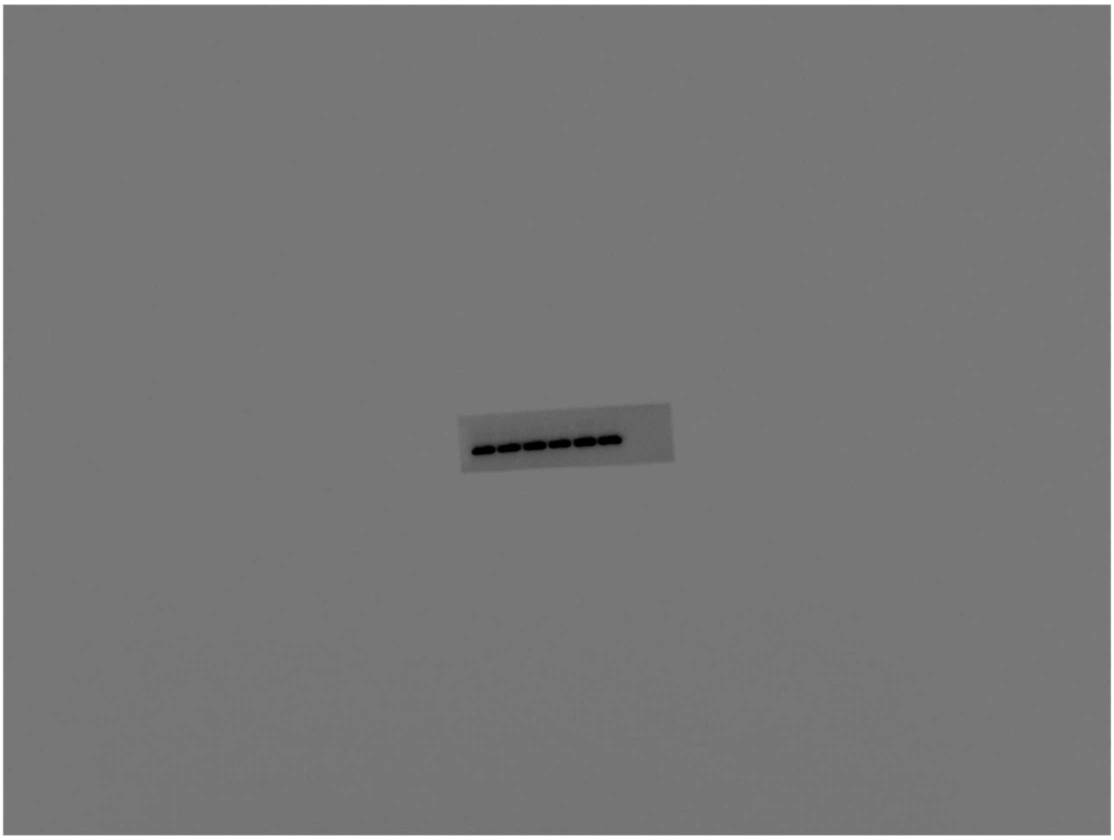

Supplement: Supplementary file 1 [file Data_Sheet_1.pdf]
